# Clinical significance and prognostic role of an immune-related gene signature in gastric adenocarcinoma

**DOI:** 10.18632/aging.203266

**Published:** 2021-07-11

**Authors:** Rui Mao, Kehao Liu, Nana Zhao, Pengsen Guo, Yingxin Wu, Zheng Wang, Yanjun Liu, Tongtong Zhang

**Affiliations:** 1 Center of Gastrointestinal and Minimally Invasive Surgery, Department of General Surgery, The Third People’s Hospital of Chengdu, Affiliated Hospital of Southwest Jiaotong University and The Second Affiliated Hospital of Chengdu, Chongqing Medical University, Chengdu 610031, China; 2 Department of Operating Room, The Third People’s Hospital of Chengdu, Chengdu 610031, China; 3 Affiliated Hospital of Southwest Jiaotong University, Chengdu 610036, China; 4 Department of Colorectal Surgery, National Cancer Center/National Clinical Research Center for Cancer/Cancer Hospital, Chinese Academy of Medical Sciences and Peking Union Medical College, Beijing 100021, China; 5 Medical Research Center, The Third People’s Hospital of Chengdu, The Affiliated Hospital of Southwest Jiaotong University, The Second Chengdu Hospital Affiliated to Chongqing Medical University, Chengdu 610031, China

**Keywords:** gastric adenocarcinoma, qRT-PCR, prognosis, immune signature, tumor microenvironment

## Abstract

Limited progress has been made in the treatment of gastric adenocarcinoma (GAC) in recent years, but the potential of immunotherapy in GAC is worthy of consideration. The purpose of this study was to develop a reliable, personalized signature based on immune genes to predict the prognosis of GAC. Here, we identified two groups of patients with significantly different prognoses by performing unsupervised clustering analysis of The Cancer Genome Atlas (TCGA) database based on 881 immune genes. The immune signature was constructed with a training set composed of 350 GAC samples from the TCGA and subsequently validated with 431 samples from GSE84437, 432 samples from GSE26253, and 145 GAC samples from real-time quantitative reverse transcription polymerase chain reaction data. This classification system can also be used to predict prognosis in different clinical subgroups. Further analysis suggested that high-risk patients were characterized by low immune scores, distinctive immune cell proportions, different immune checkpoint profiles, and a low tumor mutational burden. Ultimately, the signature was identified as an independent prognostic factor. In general, the signature can accurately predict recurrence and overall survival in patients with GAC and may serve as a powerful prognostic tool to further optimize cancer immunotherapy.

## INTRODUCTION

Despite technological advances in diagnosis and treatment, gastric adenocarcinoma (GAC) remains the most frequently diagnosed type of malignant tumor in addition to it being the primary cause of cancer-related death worldwide [1]. The global 5-year survival rates remain unsatisfactory (~25–30%) [2], except for those in Japan and South Korea (>50%) [2]. Although some factors related to tumorigenesis and prognosis, including genes [3, 4] and the tumor microenvironment (TME), have been evaluated [5], it remains mostly unclear about the precise mechanisms and signaling pathways involved. There is an urgent need to get the novel molecular biomarkers which have the ability in precisely indicating the stage of the disease progression and also predicting clinical results.

Traditional treatments for GAC include surgery, radiotherapy and chemotherapy. With the progress of medical technology, targeted therapy, angiogenic therapy and immunotherapy have become new treatments in addition to traditional therapy. As a new type of tumor therapy, immunotherapy has great potential in clinical application. Immunotherapy can achieve anti-tumor effect by acting on the patient's own immune system. With the continuous development of immunotherapy, its application in GAC has become a research hotspot. The emergence of non-specific immune enhancer therapy, immune checkpoint inhibitor therapy, adoptive immune cell therapy, oncolytic virus and tumor vaccine therapy have brought more choices and hopes to patients with GAC. Promoting the combined use of immunotherapy and other treatments, expanding the adaptation population and reducing adverse reactions can benefit more patients [6]. Immune-related genes are those genes identified through research that are significantly related to individual or partial pathways of immune response. In addition to screening the immune related genes which generate an effect on the prognosis, exploring the correlation among the immune cells, immune scores and immune checkpoints also has a certain clinical significance for the immunotherapy of GAC [7].

A comprehensive analysis in terms of the immune genes and TME in GAC and the development of a prognostic signature based on the immune gene sets (IBPS) for GAC can improve clinical risk stratification in patients with GAC and allow possible biotherapy targets to be explored. In the present study, we integrated 894 GAC patients with overall survival (OS) data and 432 patients who had recurrence-free survival (RFS) data from 5 independent cohorts, including a dataset from The Cancer Genome Atlas (TCGA), GSE84433, GSE84426, GSE26253, and 145 frozen tissue samples, for a purpose of developing and validating a novel individualized IBPS. We also performed an investigation as to the pathological characteristics, immune landscape, and also the landscape of somatic mutations in the signature.

## RESULTS

**Figure 1:**
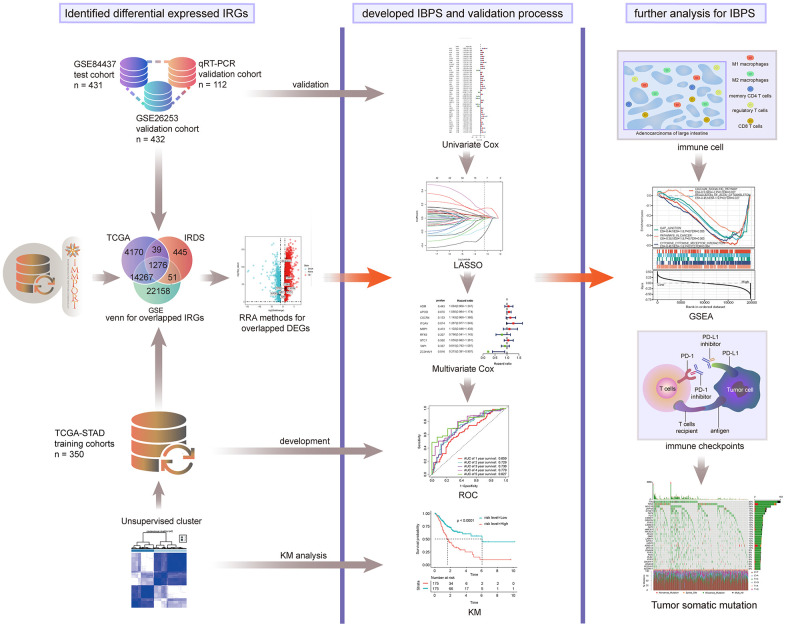
Entire analytical process of this study.

For the entire analysis process of this study, please see Figure 1. And for the clinicopathological data obtained from the TCGA, GSE84437, GSE26253, and the quantitative reverse transcription polymerase chain reaction (qRT-PCR) datasets, please see Table 1.

**Table 1:** Clinical information on patients in the training dataset, internal validation dataset and entire validation dataset.

**Characteristic**	**Training dataset** **TCGA-STAD (n=350)**	**Validation dataset** **GSE84437 (n=431)**	**Validation dataset** **GSE26253 (n=432)**	**Independent dataset (n=145)**
**Age (y)**				
**<62**	150	213	-	53
**≥62**	197	218	-	59
**Not available**	3	-	-	-
**Sex**				
**Male**	226	294	-	85
**Female**	124	137	-	27
**Survival status**				
**Alive (no recurrence)**	208	224	255	67
**Dead (recurrence)**	142	207	177	45
**pT stage**				
**T1 (T1; T1a; T1b)**	16 (5; 2; 9)	11	-	15 (0; 10; 5)
**T2 (T2; T2a; T2b)**	74 (55; 7; 12)	38	-	14
**T3**	161	92	-	2
**T4 (T4; T4a; T4b)**	95 (28; 45; 22)	290	-	81 (0; 48; 33)
**TX**	4	-	-	-
**pN stage**				
**N0**	103	80	-	47
**N1**	93	187	-	37
**N2**	72	132	-	24
**N3 (N3; N3a; N3b)**	71 (25; 40; 6)	32	-	4
**NX**	11	-	-	-
**M**				
**M0**	312	-	-	106
**M1**	23	-	-	6
**Not available**	15	-	-	-
**AJCC stage**				
**Stage I (I; IA; IB)**	46 (1; 12; 33)	-	68	26 (0; 15; 11)
**Stage II (II; IIA; IIB)**	110 (27; 34; 49)	-	167	19 (0; 4; 15)
**Stage III (III; IIIA; IIIB; IIIC)**	145 (3; 58; 51; 33)	-	111 (IIIA); 19 (IIIB)	61 (0; 37; 23; 1)
**Stage IV**	35	-	67	6
**Not available**	14	-	-	-
**Grade**				
**1**	9	-	-	6
**2**	125	-	-	29
**3**	207	-	-	77
**Not available**	9	-	-	-
**Histological type**				
**Signet ring cell type**	11	-	-	-
**Diffuse type**	61	-	-	-
**Mucinous type**	19	-	-	-
**Papillary type**	5	-	-	-
**Tubular type**	67	-	-	-
**NOS**	187	-	-	-

### Immune genes were remarkably correlated with prognosis

**Figure 2:**
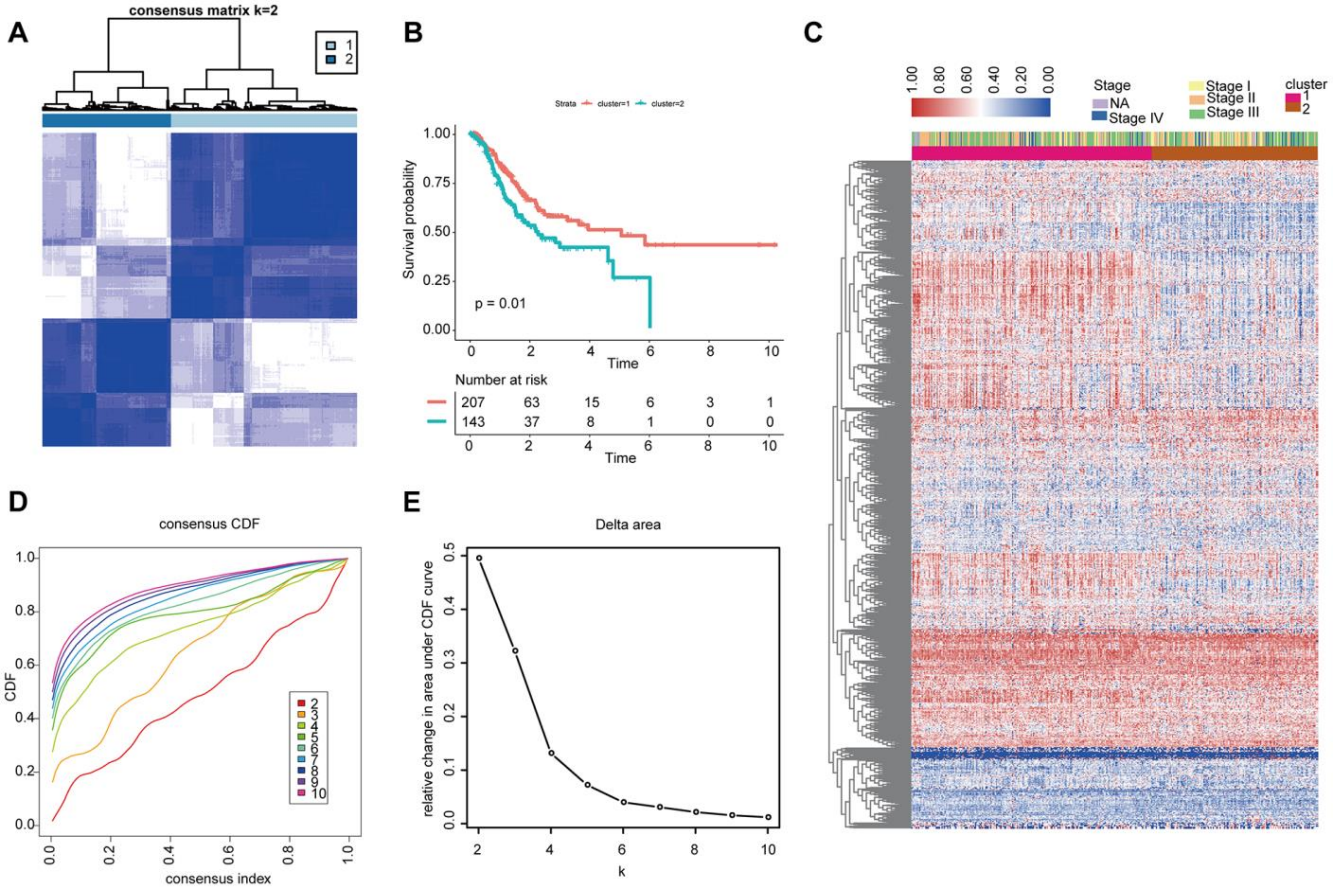
**Unsupervised clustering analysis of 881 IRGs.** (**A**, **D**, **E**) Classification of the TCGA-STAD cohort into two groups. (**C**) Landscape of the expression of 881 IRGs in the TCGA-STAD cohort. (**B**) Kaplan-Meier OS curves in the training cohort based on clusters.

First, we merged the two datasets GSE84433 and GSE84426 and removed the batch effect (Supplementary Figure 1A). Then screen the matching IRGs in the TCGA, GSE84437 and ImmPort databases (Supplementary Figure 1B), and based on the 881 differentially expressed IRGs, patients in the TCGA were classified into two groups by unsupervised clustering (Figure 2A, 2C, 2D). Prognostic analysis showed that compared with cluster-2, cluster-1 had more advantages in survival (Figure 2B).

### Differential expression analysis

We conducted a differential expression analysis as to the GAC and normal specimens from the TCGA (Supplementary Figure 1C) before we ultimately obtained 378 differentially expressed IRGs.

### The IBPS composed of 9 IRGs could effectively evaluate prognosis in the TCGA cohort

**Figure 3:**
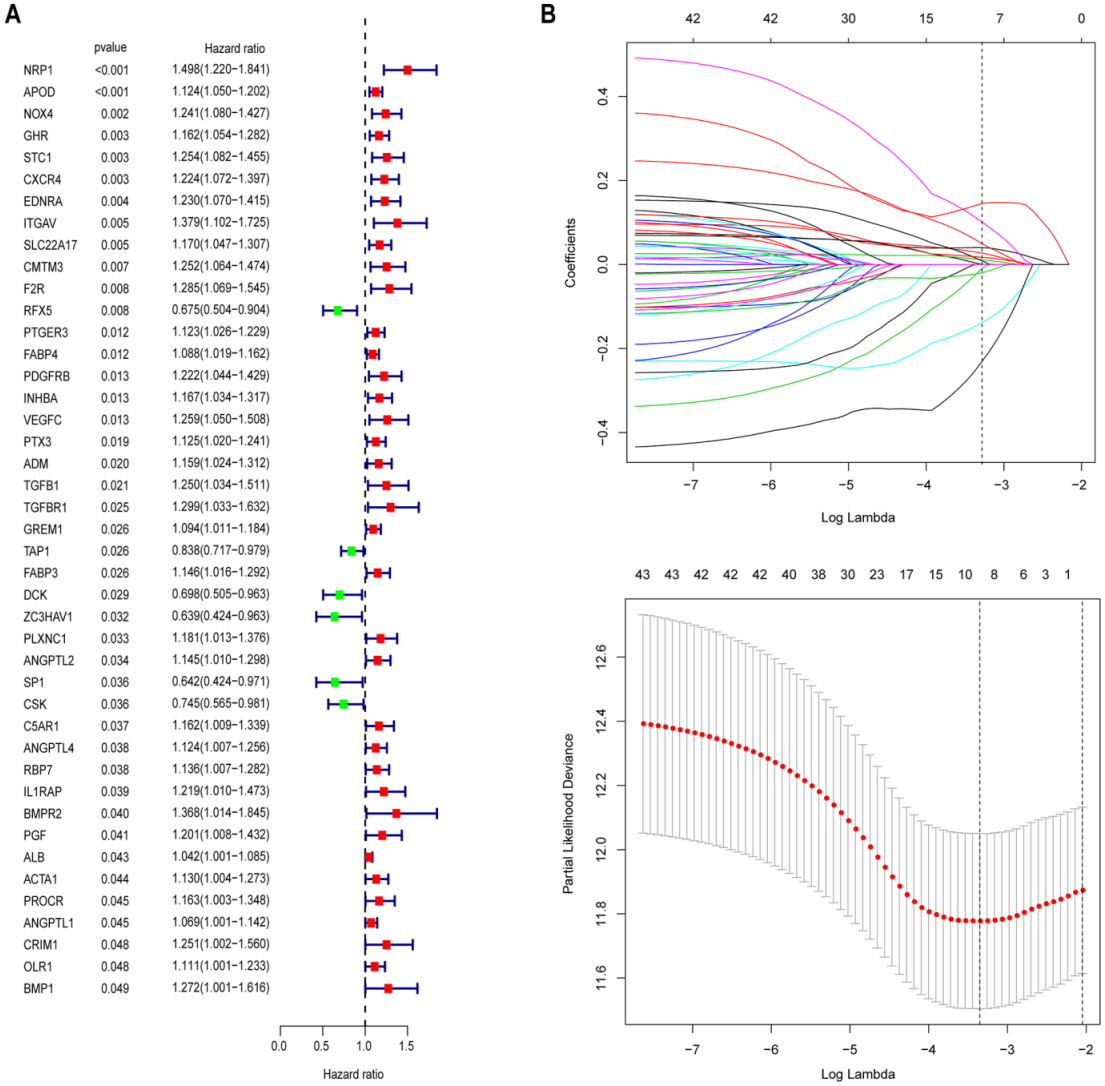
**Identification of prognostic immune-related genes in GAC.** (**A**) Univariate Cox regression analysis revealed 43 immune-related genes significantly associated with OS. (**B**) LASSO regression analysis was performed to screen the most useful prognostic genes.

**Figure 4:**
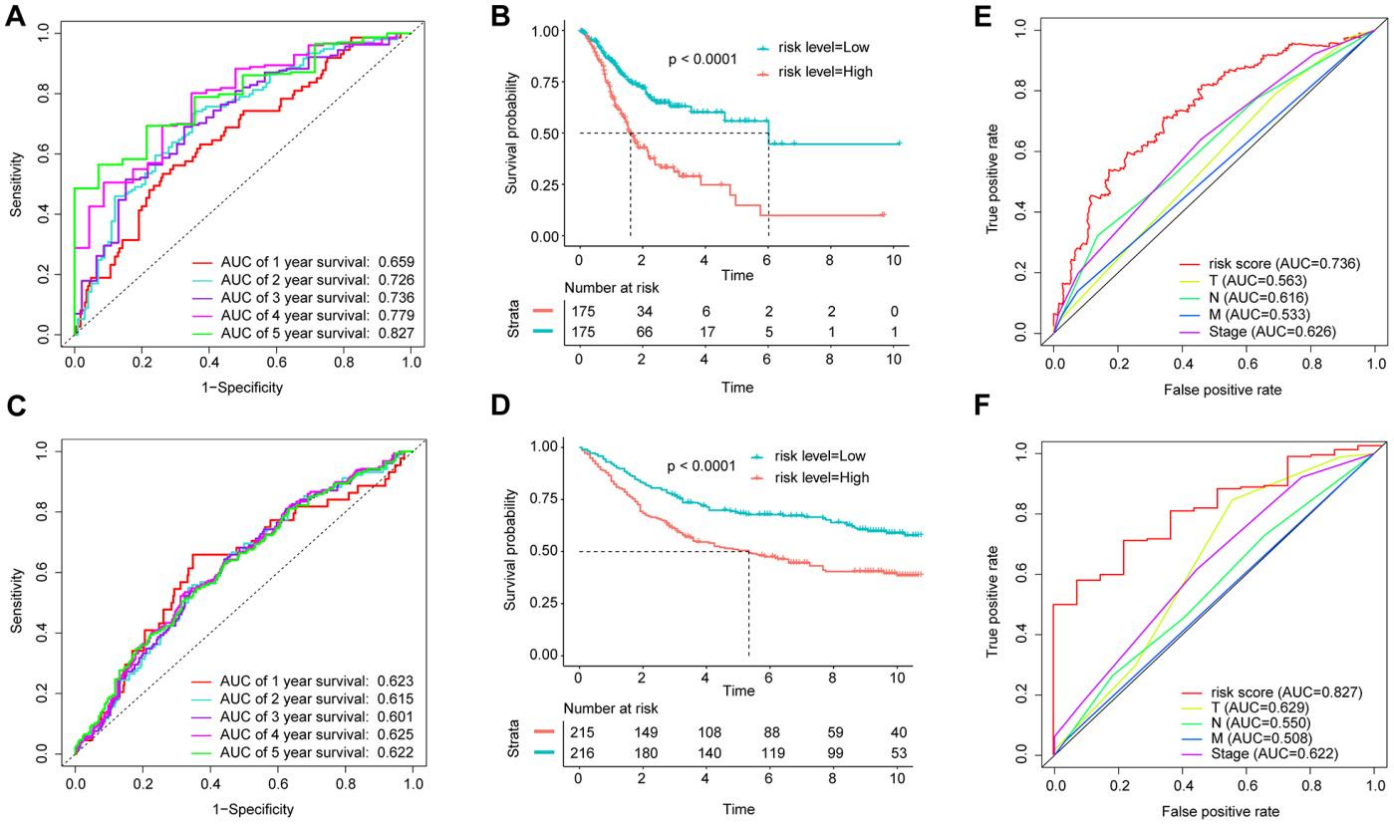
**Application of the IBPS in predicting the survival of GAC patients.** Kaplan-Meier survival curves of the hub RNAs in the ceRNA network. (**A**) ROC curve analysis of the immune-related gene signature for the prediction of OS at 1, 2, 3, 4, and 5 years in the TCGA cohort; (**B**) Kaplan-Meier curves of OS in all GAC patients in the TCGA cohort based on the risk score; (**C**) ROC curve analysis of the immune-related gene signature and TNM stage for the prediction of OS at 3 years in the TCGA cohort; (**D**) ROC curve analysis of the immune-related gene signature for the prediction of OS at 1, 2, 3, 4, and 5 years in the GEO cohort; (**E**) Kaplan-Meier curves of OS in all GAC patients in the GEO cohort based on the risk score; (**F**) ROC curve analysis of the immune-related gene signature and TNM stage for the prediction of OS at 5 years in the TCGA cohort.

Through a univariate Cox survival analysis, there were 43 IRG with P < 0.05 chosen from 378 IRG for follow-up analysis (Figure 3A). Figure 3B indicated that via the least absolute shrinkage and selector operator (LASSO) regression analysis, there were 9 IRGs identified (“lambda.min” criteria). The results of multivariate COX analysis of the 9 IRGs are shown in Supplementary Table 2. Ultimately, 9 IRGs predictive of GAC patient survival, namely, ADM, APOD, CXCR4, ITGAV, NRP1, RFX5, STC1, TAP1, and ZC3HAV1, were identified. The formula of the IBPS was calculated: risk score = (0.0622 × exp of ADM) + (0.0771 × exp of APOD) + (0.1335 × exp of CXCR4) + (0.2369 × exp of ITGAV) + (0.0968 × exp of NRP1) - (0.2405 × exp of RFX5) + (0.0531 × exp of STC1) - (0.0892 × exp of TAP1) - (0.5188 × exp of ZC3HAV1). Via taking the median risk score as the cut-off point, a division of the patients in the training set into high-risk group and low-risk group was conducted (Supplementary Figure 3A). The expression levels of the 9 IRGs are shown in Supplementary Figure 2. The patients in the low-risk group had a significantly better OS than the high-risk group (Figure 4B). Besides, there was an analysis of the receiver operating characteristic (ROC) performed, for an evaluation of the IBPS's prediction of the survival in terms of its accuracy (Figure 4A). The results suggest that there is an area of 0.736 (3 years) and 0.827 (5 years), under the curve (AUC value) of IBPS, respectively, which is larger than the AUC value of the system of the TNM pathological staging (Supplementary Figure 4E).

### The IBPS could effectively evaluate prognosis in the GSE84437 cohort

In order to validate the robustness of IBPS, we also analyze it with GSE84437 dataset (n = 431). The results suggest that it can also predict the prognosis in GSE84437 (Supplementary Figures 3B, 4C, 4D).

### The IBPS could effectively evaluate prognosis in different clinical subgroups

In general, patients in the low-risk group had better OS than those in the high-risk group according to different pathological stages (Supplementary Figure 4A–4C). In the stage IV subgroup (P = 0.19) (Supplementary Figure 4D), we could clearly see the same trend, but it was not statistically significant, potentially due to insufficient sample sizes.

Although the pathological stage is one of the factors that most influences survival of GAC, other factors, such as age, grade, sex, and major histological phenotype, are also contributors [8]; therefore, we grouped the GAC patients in the TCGA training cohort according to the above clinical features. According to the results, in all subgroups (older (aged ≥ 62) and younger (aged < 62), grade 2 and grade 3, males and females, and intestinal type and diffuse type), the low risk groups had better OS than the high risk groups (Supplementary Figure 5, P < 0.05). Predictably, validation of these results was observed in the GSE84437 dataset (Supplementary Figure 6). Additionally, the WHO provides detailed descriptions of histological features and classifies GAC into tubular, papillary, mucinous (MUC), mixed, and signet ring cell (SRC) types. We analyzed the performance of the IBPS among these subtypes. The results showed a notable concentration of the SRC type in high-risk scores group (Supplementary Figure 7A). Among patients with the tubular, MUC or papillary subtype, there was significantly shorter OS observed in the high-risk patients compared with the low-risk patients (Supplementary Figure 7B–7D, P < 0.05). Besides, among all GAC patients who received chemotherapy, there was longer OS time observed in the patients in the low risk groups compared with the high risk groups (Supplementary Figure 7E, P < 0.0001). According to the results presented in Supplementary Figure 7F, 7G, there was significantly worse chemotherapy effect observed in the patients with high risk score (3-year OS rate: 35%) compared with the patients with low risk score (3-year OS rate: 70%).

### The IBPS could effectively evaluate prognosis in the GSE26253 cohort

For an exploration of whether the IBPS can equally work in prediction of the RFS outcome of GAC patients, a similar analysis was performed for the GSE26253 cohort (n = 432, Supplementary Figure 8A, 8B). The results showed that the IBPS could effectively evaluate the RFS outcome of GAC patients. Besides, we observed that the RFS duration of patients with advanced-stage disease was significantly shorter than that of patients with early-stage disease (Supplementary Figure 8C). It is worth noting that in pathological stage I and stage II subgroups, better RFS was seen in the patients in the low-risk groups compared with the high-risk groups (Supplementary Figure 8D, 8E, P <0.01).

### The IBPS could effectively evaluate prognosis in the qRT-PCR group

**Figure 5:**
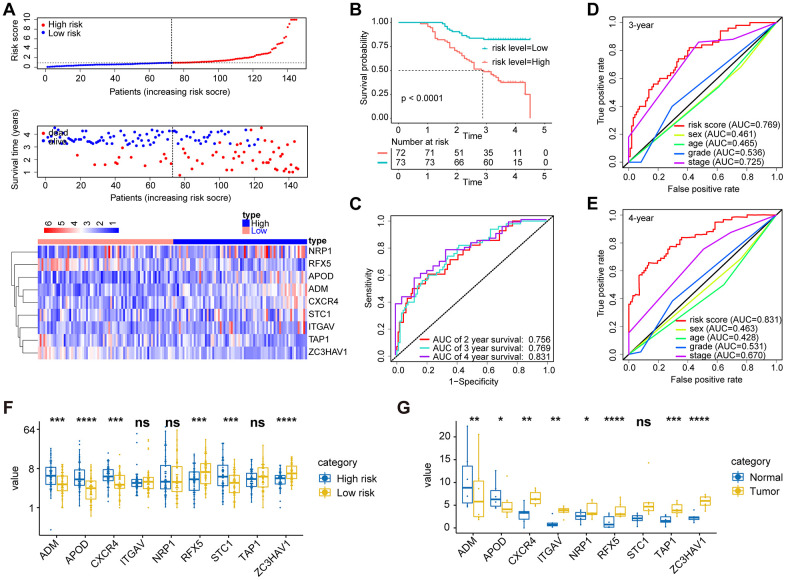
**Validation of the prognostic performance of the immune-related gene signature in an independent group based on 145 frozen tissues.** (**A**) Distribution of the risk score, survival status, and gene expression panel. (**B**) Kaplan-Meier curves of OS in all GAC patients based on the risk score. (**C**) ROC curve analysis of the immune-related gene signature for the prediction of OS at 2, 3, and 4 years in the independent cohort. (**D**) ROC curve analysis for the prediction of OS at 3 years in an independent cohort based on multiple clinical features. (**E**) ROC curve analysis for the prediction of OS at 4 years in an independent cohort based on multiple clinical features. (**G**) The expression of 9 immune genes in gastric cancer tissues and their corresponding normal tissues. (**F**) We also compared the differential expression of 9 immune genes between the high- and low-risk groups.

For a validation of the IBPS in terms of its robustness, we conducted the same analysis with the qRT-PCR validation cohort (n = 145) (Figure 5A, 5B). The AUCs for the prediction of 3- and 4-year survival by the IBPS reached 0.769 and 0.831, respectively (Figure 5C), which were larger compared to the system of the traditional TNM pathological staging (Figure 5D, 5E). Furthermore, when applied to subcategories of patients with GAC in different pathological stages at the time of diagnosis and other different clinical subgroups, the risk score was predictive of significantly different OS outcomes (Supplementary Figure 9, P < 0.05). We compared the expression of 9 IRGs in gastric cancer tissues to their corresponding normal tissues (Figure 5F). We also compared the differential expression of 9 IRGs between the two groups (Figure 5G).

### Patients in the High-risk group had the below features, namely, low immune scores, distinctive immune cell proportions, and different immune checkpoint profiles

To explore the potential mechanism between the IBPS and OS in GAC patients, we performed multiple analyses related to the immune profile. The stromal, immune, and ESTIMATE scores showed significant differences between the two groups (Supplementary Figure 10A, 10B). Kaplan-Meier analysis of data on GAC patients in the TCGA cohort showed that different stromal and ESTIMATE scores produced differential OS outcomes (Supplementary Figure 10C, 10E). And, we obtained similar results in the GSE84437 cohort (Supplementary Figure 10D, 10F).

Since there was a close relation of the risk score with the immune infiltration score, we analyzed the high-risk group and the low-risk group in terms of their differences in immune cell infiltration and immune checkpoints. The results obtained from the TCGA and Gene Expression Omnibus (GEO) datasets are shown in Supplementary Figure 11A. M1 macrophages, M0 macrophages, memory CD4 T cells, M2 macrophages, and CD8 T cells constituted a large proportion of the GAC-infiltrating immune cells. Besides, the two group showed most of the differential immune cells, which were predominantly M1 macrophages, monocytes, CD8 T cells, follicular helper T cells, memory CD4 T cells, and M2 macrophages (Supplementary Figure 11B, 11C). Furthermore, as observed, the 9 IRGs had a Pearson correlation with varied immune cells (Supplementary Figure 12). We also compared the differences in 49 immune checkpoints, and the results obtained from the TCGA and GEO datasets are shown in Supplementary Figure 13A, 13B, respectively. Overall, the expression of VTCN1, ENTPD1, and FGL1 was obviously upregulated while that of LGALS9 was dramatically downregulated in the high-risk group of patients in both cohorts (Supplementary Figure 14, P < 0.05). Besides, good correlations were observed between the 9 IRGS and various differentially expressed immune checkpoints, especially those for TAP1 and CXCR4 (Supplementary Figure 15).

### Patients in the High-risk group were characterized with a low tumor mutational burden (TMB)

**Figure 6:**
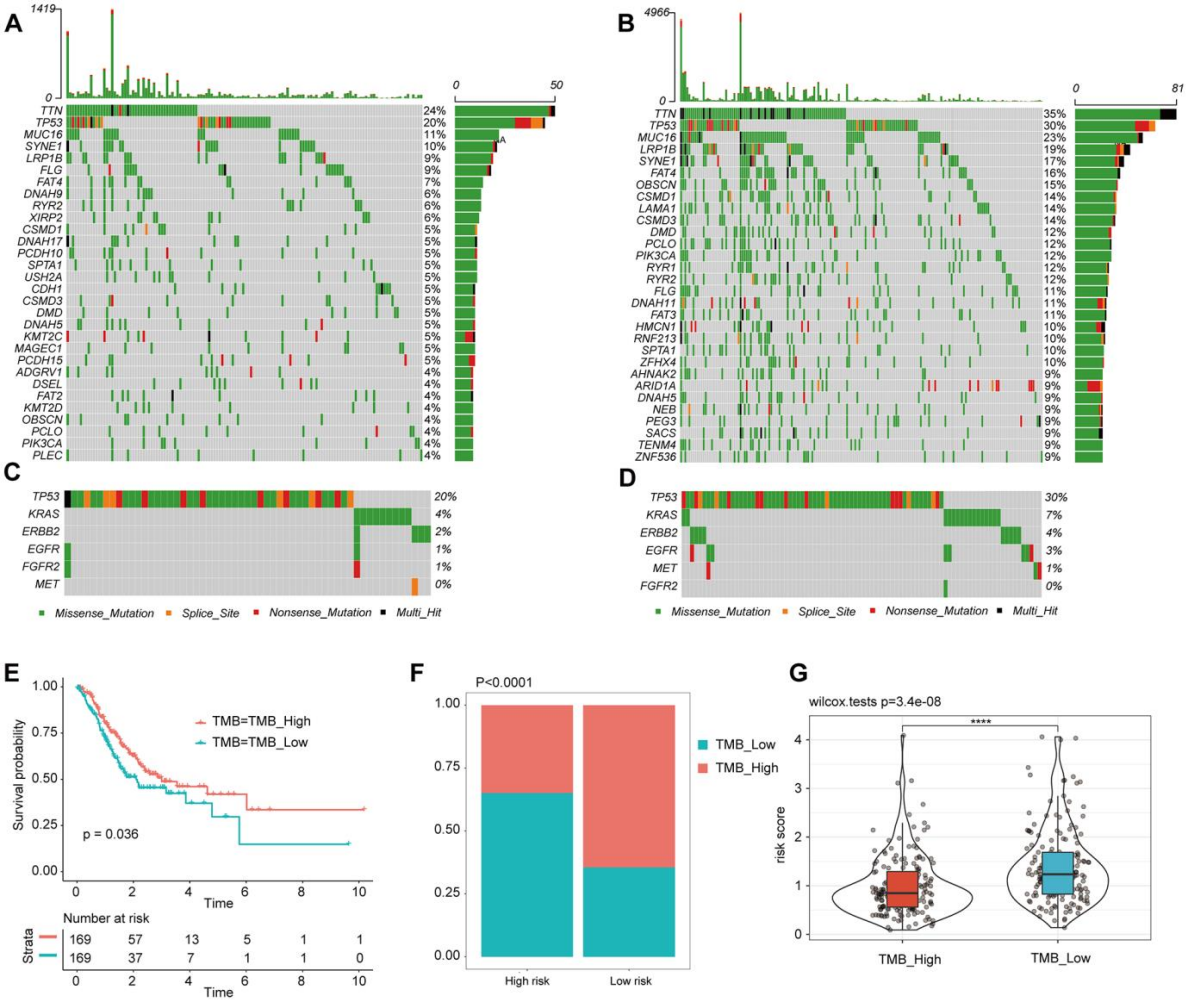
**Tumor somatic mutational landscape of the IBPS.** (**A**) The top 35 gene mutations in the high-risk score group. (**B**) The top 35 gene mutations in the low-risk score group. (**C**, **D**) Mutations in the commonly mutated genes TP53, ERBB2, EGFR, FGFR2, MET, and KRAS in the high-risk and low-risk score groups, respectively. (**E**) Kaplan-Meier curves of OS in all GAC patients based on TMB. (**F**) Difference in TMB between the high- and low-risk groups. (**G**) Difference in risk scores between the high and low TMB groups.

Then, we used the maftools software package to analyze the difference in the distribution of somatic mutations between the low-risk score group and the high-risk score group in the TCGA-STAD cohort. Supplementary Figure 16A shows the top 30 gene mutations in the TCGA-STAD cohort. The top 30 gene mutations in the high-risk score group and the low-risk score group are shown in Figure 6A, 6B, respectively. Somatic mutations were altered in 355 of 427 samples (83.14%). The well-known oncogenes TP53, ERBB2, EGFR, FGFR2, MET, and KRAS [9] in GAC were altered in 182 of 427 samples (42.62%) (Supplementary Figure 16B), especially TP53 (Figure 6C, 6D). Kaplan-Meier curve analysis suggests that patients with a high TMB have significantly better OS than patients with a low TMB (Figure 6E). And there was lower TMB observed in the high risk score group compared to the low risk score group (P < 0.0001, Figure 6F). What's more, the high TMB group had lower risk scores compared to the low TMB group **(**Figure 6G). Besides, we explored that the mutational rates of the 9 IRGs in the IBPS were very low (Supplementary Figure 16C), supporting their use as diagnostic or prognostic biomarkers.

### The IBPS is an independent risk factor for GAC patients

Through univariate and multivariate Cox regression analysis of various clinical factors in the TCGA cohort (Table 2), we screened out the independent prognostic risk factors such as age, histological type, TNM stage, IBPS and so on. The specific variables significantly related to survival included age > 62 years (hazard ratio (HR)=1.89, P< 0.001), the MUC type (HR=0.25, P= 0.02), stage IV (HR=3.86, P=0.0005), stage III (HR=2.28, P=0.017), and a rising risk score (HR=2.31, P<0.0001). Besides, the results obtained from the GSE84437 cohort confirmed the value of the IBPS for OS (P = 0.0037) in GAC patients (Table 3**)**. Additionally, Supplementary Table 3 showed that the IBPS was also an independent risk factor for RFS (P=0.0300). As expected, there were similar results observed in the qRT-PCR cohort (Supplementary Table 4**)**.

**Table 2:** Univariable and multivariable Cox regression analyses of the IBPS and correlations with OS in the TCGA cohort.

**Variable**	**Overall survival**						
	**Univariate Cox**				**Multivariate Cox**		
	***p* value**	**HR**	**95% *CI***		***p* value**	**HR**	**95% *CI***
**Age**							
≥62 vs <62	0.0075	1.6050	1.1350-2.2700		0.0009	1.8900	1.2976-2.7528
**Sex**							
Male vs Female	0.1760	1.2780	0.8954-1.8250		0.4200	1.1630	0.8055-1.6800
**Histological type**							
NOS							
Signet ring cell type	0.0565	2.0307	0.9806-4.2054		0.6783	1.1780	0.5438-2.5505
Diffuse type	0.2685	0.7689	0.4827-1.2247		0.1812	0.7087	0.4278-1.1741
Mucinous type	0.0152	0.2395	0.0755-0.7592		0.0206	0.2520	0.0785-0.8094
Papillary type	0.5446	1.4284	0.4507-4.5277		0.1640	2.4470	0.6940-8.6276
Tubular type	0.2558	0.7702	0.4910-1.2083		0.7323	0.9184	0.5640-1.4957
**TNM stage**							
I							
II	0.1857	1.5830	0.8017-3.1280		0.1553	1.6560	0.8260-3.3188
III	0.0144	2.2300	1.1734-4.2380		0.0170	2.2790	1.1584-4.4817
IV	0.0004	3.6980	1.7921-7.6310		0.0005	3.8550	1.7978-8.2680
NA	0.0002	5.1930	2.1954-12.2840		0.0008	4.7050	1.9007-11.6448
**Grade**							
1							
2	0.4690	1.6870	0.4097-6.9480		0.2812	2.2500	0.5148-9.8341
3	0.2910	2.1300	0.5240-8.6590		0.1229	3.2190	0.7289-14.2170
NA	0.2280	2.8420	0.5201-15.5360		0.0662	5.4690	0.8923-33.5155
**Risk score**							
Increasing	<0.0001	2.4650	1.7420-3.4880		<0.0001	2.3050	1.5836-3.3562

IBPS, immune gene set-based prognostic signature; HR, hazard ratio; CI, confidence interval.

**Table 3:** Univariable and multivariable Cox regression analyses of the IBPS and correlations with OS in the GSE84437 cohort.

**Variable**	**Overall survival**						
	**Univariate Cox**				**Multivariate Cox**		
	***p* value**	**HR**	**95% *CI***		***p* value**	**HR**	**95% *CI***
**Age**							
≥62 vs <62	0.0022	1.5387	1.1670-2.0290		0.0007	1.6171	1.2235-2.1370
**Sex**							
Male vs Female	0.1660	1.2395	0.9151-1.6790		0.2367	1.2038	0.8854-1.6370
**T stage**							
1							
2	0.9463	0.9474	0.1968-4.5610		0.7378	0.7624	0.1577-3.7330
3	0.1904	2.5859	0.6238-10.7200		0.4621	1.7180	0.4016-7.2680
4	0.0547	3.9247	0.9729-15.8330		0.2187	2.4322	0.5900-10.0260
**N stage**							
0							
1	0.1020	1.4487	0.9287-2.2600		0.2378	1.3131	0.8354-2.0640
2	<0.0001	2.9315	1.8826-4.5650		0.0004	2.2971	1.4547-3.6270
3	<0.0001	3.7702	2.1294-6.6750		0.0027	2.4691	1.3683-4.4550
**Risk score**							
Increasing	<0.0001	1.8140	1.3730-2.3970		0.0037	1.5264	1.1474-2.0310

IBPS, immune gene set-based prognostic signature; HR, hazard ratio; CI, confidence interval.

In addition, an investigation was performed as to the IBPS in terms of its comprehensive prognostic value in all groups by prognostic meta-analysis (n = 893). According to the results, the IBPS was a significant risk factor for OS in GAC patients (combined HR = 2.218, 95% CI = 1.804–2.727, P < 0.0001) (Supplementary Figure 17).

### Multiple immune and tumor-related pathways associated with the IBPS

GSEA identified 20 significant KEGG pathways associated with the risk score, including cell adhesion molecules (CAMs), the MAPK signaling pathway, DNA replication, nucleotide excision repair, the cell cycle, cytokine-receptor interaction, the P53 signaling pathway, mismatch repair, ECM-receptor interaction, and pathways in cancer (Supplementary Figure 18).

## DISCUSSION

GAC accounts for approximately 95% of the histological types of all malignant tumors that originate in the stomach. Although patients with early-stage GAC (stage I) have a five-year survival rate of 95% [10], the median survival time of patients with advanced-stage GAC, which cannot be treated by surgery, is approximately 9-10 months [11]. It is urgently necessary to find out reliable early screening methods for identifying early GAC. In this study, we performed an unsupervised clustering analysis of 350 GAC patients from the TCGA before we made a confirmation that there was a remarkable correlation of the immune genes with the OS. Nine immune genes (ADM, APOD, CXCR4, ITGAV, NRP1, RFX5, STC1, TAP1, and ZC3HAV1) were applied to construct a prognostic signature for GAC. The 431 GAC patients in the GSE84437 cohort were used to validate the stability of the IBPS. To avoid false positives in sequencing data, another verification was performed based on the qRT-PCR results of 145 frozen tissue samples from GAC patients, confirming our previous findings and evaluating the utility of this signature in the Chinese population. In addition, by analyzing the GSE26253 dataset, we identified that the combination of IRGs can also be used to accurately predict recurrence in GAC patients. We found a report that established a signature of GAC based on immune cells and related genes [12], but compared with previous signatures, our signature has some novelty: (1) The ability of our signature (AUC = 0.827) in predicting the 5-year OS rate of patients with GAC is higher than that of Qiu et al [13] (AUC = 0.761), Peng [14] (AUC = 0.737), Yang [15] (AUC = 0.802), and Peng [16] (AUC = 0.728). (2) Our signature can accurately predict survival and recurrence at the same time. (3) The signature includes only 9 immune genes instead of 14 [17], making it easier for us to implement our model. (4) A validation cohort based on the qRT-PCR was employed for ensuring the robustness of our signature, and all the immune genes contained in the signature were confirmed by qRT-PCR to be significantly differentially expressed in GAC and corresponding normal tissues, which further confirms the accuracy of our signature. (5) We found little research on TAP1 and APOD in the context of GAC. These molecules may serve as therapeutic targets for gastric cancer.

Besides, for different Patients with various stages of GAC, their immune statuses are different. Their responses to immunotherapy are different, too [18] we identified that the ability of IBPS to predict the prognosis of GAC is independent of pathological stage. Histological phenotypes and tumor differentiation grade are closely associated with prognosis and the tumor immune microenvironment [18]. As expected, the IBPS performed stably in all differentiation/grade subgroups and tumor subtypes. Additionally, we analyzed the performance of the IBPS among the histological subtypes of GAC, which include mixed, papillary, MAC, tubular, and SRC types [8]. In all subtypes except for SRC, there was significantly better OS observed in the low-risk group compared with the high-risk group. According to reports, there were shorter OS times observed in the patients with the SRC and MAC subtypes compared to those with the other subtypes [19, 20]. Interestingly, as we observed, there was significantly lower average risk score in the GAC patients with the SRC and MAC subtypes compared to the GAC patients with the other two subtypes, possibly confirming our signature in terms of its favorable prognostic value. Moreover, in the qRT-PCR cohort, the IBPS was used to perform an accurate risk stratification among the four subtypes of GAC (Supplementary Figure 19). In addition, the patients with high risk score (3-year OS rate was 35%) had significantly worse chemotherapy effect compared to the patients with low risk score (3-year OS rate was 70%). In order to better guide immunotherapy, we applied this signature to the data of skin melanoma in TCGA to test its efficacy. According to the results, there was worse immunotherapeutic effect on cutaneous melanoma patients with high risk scores compared to those with low risk scores (5-year OS: 51% vs 85%, Supplementary Figure 20B), and the AUC of the signature for predicting the 3-year survival of patients who received immunotherapy reached as high as 0.877 (Supplementary Figure 20A). Besides, we found that in the anti-CTLA-4 immunosuppressive therapy cohort (GSE63557) and the anti-MAGE-A3 immunosuppressive therapy cohort (GSE35640), the non-responders to immunotherapy had significantly higher risk scores compared to the responders (Supplementary Figure 20C, 20D). This signature may have great significance in guiding stratified treatment in the clinic. The intensity of treatment can be adjusted down for low-risk patients, who should consider adjuvant immunotherapy and chemotherapy before and after surgery. There is a need for the high-risk patients to think about surgical total resection more actively and undergo frequent checkups to monitor recurrence and allow any further treatment to be carried out promptly.

Age and sex also work on the prognosis of GAC patients [21]. The incidence of GAC is approximately two times higher in men than in women [22]. Compared with male patients, female patients with gastric cancer (GC) were found to be significantly younger. The signature had a convincingly well performance in all subgroups. These findings further convince us on our signature's ability in identifying high-risk patients in any clinical subgroup of GAC patients and better guide clinical treatment.

VTCN1, ENTPD1, and FGL1 levels were obviously upregulated while the LGALS9 level was dramatically downregulated in the high-risk group of patients. VTCN1, also called B7-H4, is a vital immune checkpoint molecule, and a member of the B7 family [23]. B7-H4 may promote gastric cancer progression by inhibiting the antitumor immune response. Cai et al found that the overexpression of ENTPD1 in patients with GAC predicted a poor outcome [24]. Research has also shown that fibrinogen-like-protein 1 (FGL1) expression is upregulated in GC tissues and that OS is significantly shorter in patients with high FGL1 expression compared to those with low FGL1 expression. In addition, *in vitro* tests have shown that FGL1 promotes the invasion and metastasis of gastric cancer cells [25]. Upregulation of LGALS9, also known as galectin-9, can inhibit cell invasion, migration, and epithelial–mesenchymal transition (EMT) in intestinal-type gastric cancer [26]. According to this study, combinations of immune genes may activate or inhibit the development of GAC by inhibiting or increasing the expression of these immune checkpoints. To further verify this possibility, we performed a correlation analysis of 9 IRGs and immune checkpoints in each cohort. The results showed a strong correlation between the IRGS and immune checkpoints, especially TAP1 and CXCR4.

There have been a large number of studies on a variety of tumors showing that patients with a high TMB tend to enjoy good survival rates [27]. In the present study, patients with a high TMB have significantly better OS than patients with a low TMB. And there was lower TMB seen in the high-risk score group compared to the low-risk score group. As observed, most of the genes in the IBPS were involved in tumor immune microenvironment remodeling and tumor progression [23, 28–32]. Previous studies have demonstrated that CXCR4 can contribute to EMT, migration, and invasion in gastric cancer through immune and inflammatory pathways [33]. Both TAP1 and APOD are closely related to antitumor immunity, and studies have shown that TAP1 plays an important role in a variety of cancers [34–36]. It is worth noting that research on TAP1 and APOD in the context of GAC is lacking, and thus, these molecules require further exploration.

Overall, this study offers new insights into the correlation of the immunotherapy with GAC, which could better guide the clinical treatment of GAC.

## MATERIALS AND METHODS

### Data acquisition and preprocessing

Training sets, including the mRNA expression profiles with FPKM format of GAC specimens and the corresponding clinical follow-up data, were downloaded from the TCGA (https://portal.gdc.cancer.gov/) database. The data employed in this study were in line with the below criteria: (1) mRNAs with nonzero expression levels accounted for 75% of all samples; and (2) the patients had exact follow-up times. After excluding 24 patients with an OS time of 0, 5 without OS data, and 64 without RNA expression matrix information, a total of 350 gastric cancer patients remained. Thirty-two normal gastric specimens from the TCGA database were also included.

The GSE84437 and GSE26253 datasets, which were regarded as the validation cohort, were downloaded from the GEO (https://www.ncbi.nlm.nih.gov/gds/) database and were first log2 transformed and quantile normalized. The GSE84437 dataset consists of the two subsets GSE84433 and GSE84426. The combination function of R software package "SVA" is used for eliminating the batch effect when combining GSE84433 and GSE84426 data sets. After excluding 2 patients with an OS time of 0, 431 GAC specimens were ultimately included. The GSE26253 dataset contains 432 GAC patients with RFS data.

In addition, 145 samples were obtained from pathologically confirmed GAC patients between June 2012 and August 2014 at the Colorectal Surgery Department of National Cancer Center.

We retrieved and downloaded the IRG list from the ImmPort database (https://immport.niaid.nih.gov).

### Unsupervised clustering analysis of IRGs in the TCGA

We use the mRNA matrix data downloaded from the TCGA and GEO databases and the IRGS to intersect. The patients were analyzed by unsupervised cluster analysis according to the expression of IRGS. The consistent clustering algorithms were adopted for determining the clusters in terms of their quantity and stability [37]. We performed the above analysis using the ConsensusClusterPlus package [38] and repeated the analysis for 1000 times for ensuring the classification stability.

### Differential analysis

The edgeR package [39] was used to analyze the difference in coincident IRGs in 350 gastric cancer tissues and 32 normal tissues from the TCGA.

### Evaluation of prognosis by IBPS

We use R packages "glmnet," "survminer," and "survival." to perform the univariate and multivariate COX regression analysis. The ROC curves and the corresponding AUC were generated with the R package “survivalROC”.

### Exploration of the relationships between the IBPS and immunity or the TME

The immune score of each sample was determined by R software using estimation algorithm, and the difference of immune score between high risk group and low risk group was further compared with Wilcoxon test [40]. The proportion of 22 immune cell subtypes was evaluated by CIBERSORT software package according to the expression profile [41]. The difference of immunocyte subtypes between high-risk and low-risk groups was analyzed by Mann-Whitney U test. 49 immune checkpoint included the B7-CD28 family (TMIGD2, CD274 (PD-L1), ICOS, PD-1, B7-H3, CTLA4, PD-L2, ICOSLG, and HHLA2) [20, 42], the TNF superfamily (CD40LG, TNFRSF18, TNFRSF4, TNFSF4, TNFRSF25, TNFRSF14, CD27, TNFRSF8, CD40, TNFSF15, TNFSF14, TNFSF9, TNFRSF9, TNFSF18, and CD70) [43], and several other immune checkpoint members (CD244, CD44, IDO1, CD160, IDO1, TIGIT, CD200, KIR3DL1, LAG3, LAIR1, CD80, CD28, NRP1, NCR3, CD48, ENTPD1, FGL1, HAVCR2, BTNL2, CD86, IDO1, IDO2, and ADORA2A)) [44–46].

### GSEA

Based on the software GSEA v4.0.3, we performed GSEA (http://www.broadinstitute.org/gsea). We input the expression profile of the mRNAs, group of samples, and enriched background file.

### qRT-PCR

The extraction, reverse transcription and amplification of RNA refer to the methods in our previous study [47]. Supplementary Table 1 provides the primers used in this study.

### Statistical analysis

Correlation coefficients between TME-infiltrating immune cells, immune checkpoints, and the expression of IRGs were computed by Pearson analyses. One-way ANOVA and Kruskal–Wallis tests were used to assess differences between three or more groups [48, 49].

### Data availability statement

The data obtained from the TCGA and GEO datasets in this study are publicly available. Our research has been approved by the Institute of Oncology / Hospital Ethics Committee / Institutional Review Committee of Peking Union Medical College and the Chinese Academy of Medical Sciences (approval no. NCC2013RE-025).

## Supplementary Material

Supplementary Figures

Supplementary Tables
