# Supplementary material for: Clinical significance and prognostic role of an immune-related gene signature in gastric adenocarcinoma
Source: Aging (Albany NY). 2021 Jul 11;13(13):17734–67. doi: 10.18632/aging.203266 (PMC8312416; doi:10.18632/aging.203266)
Supplement: Supplementary Figures [file aging-13-203266-s001.pdf]

SUPPLEMENTARY FIGURES

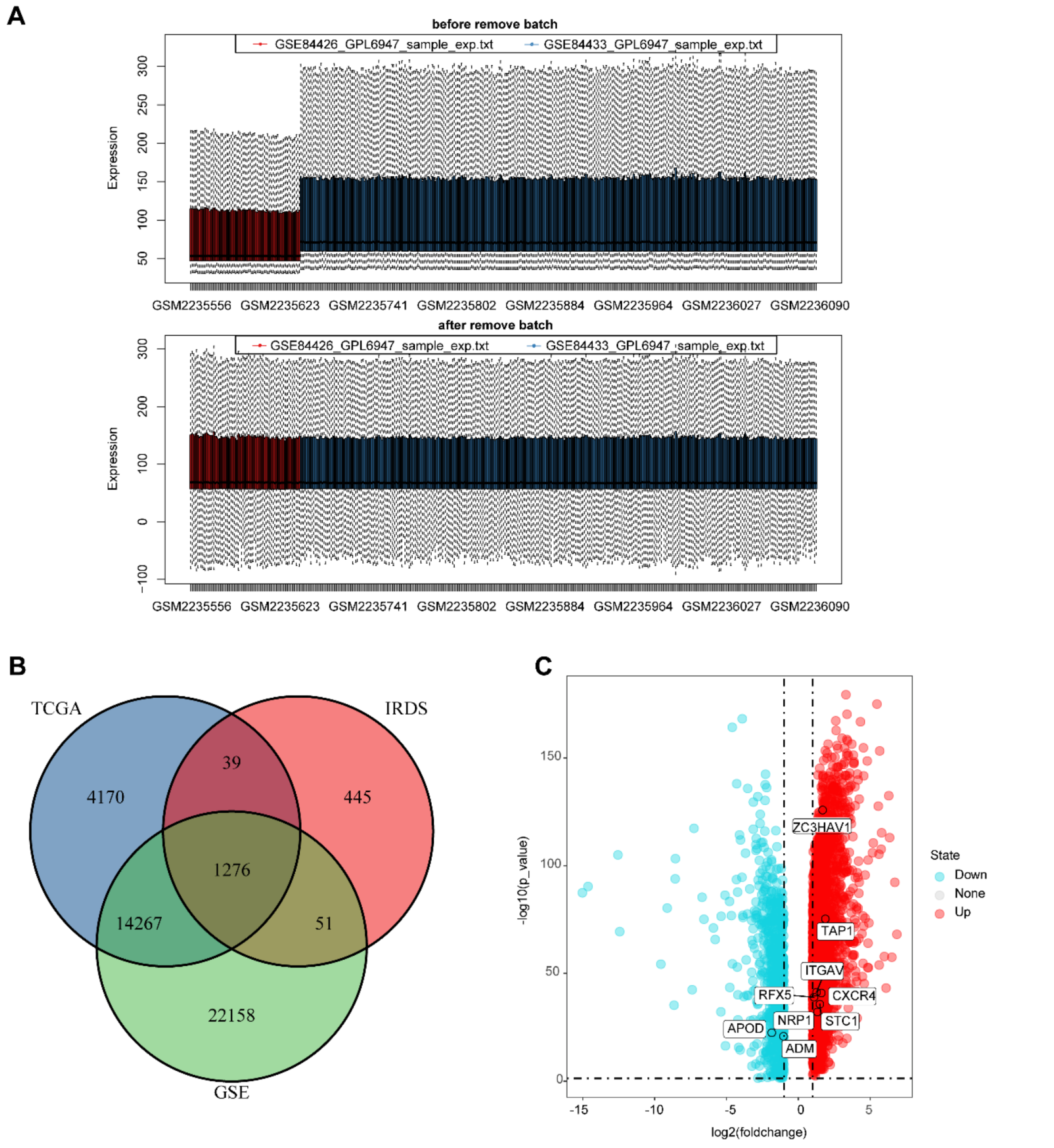

Supplementary Figure 1. Data processing and screening differences IRGS between stomach adenocarcinoma and para-carcinoma tissues. (**A**) The boxplot shows that we have merged the two datasets GSE84433 and GSE84426 and removed the batch effect. Then, as the Venn diagram shows, IRGS coincident in the TCGA, GSE84437, and ImmPort databases were screened. (**B**) The difference analysis was performed in the TCGA gastric adenocarcinoma and normal specimens. (**C**) Red indicates high expression and blue indicates low expression.

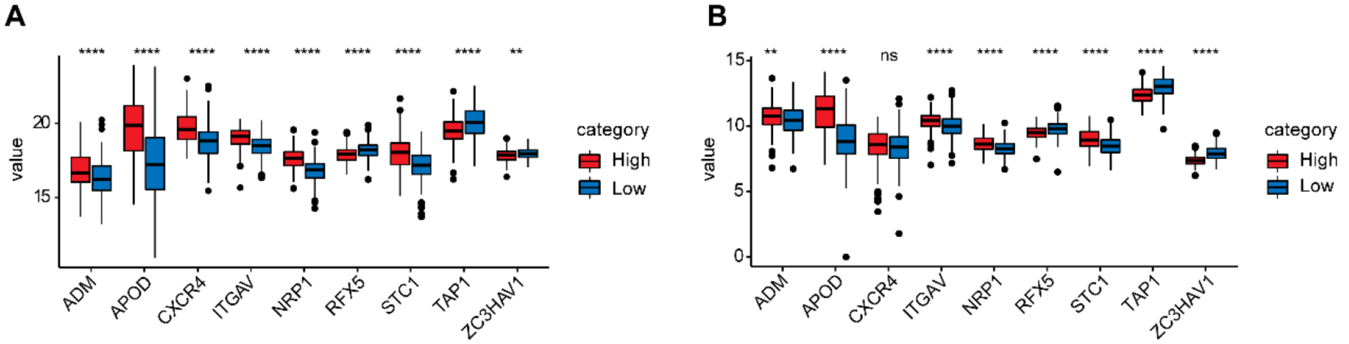

Supplementary Figure 2. Different expression of immune genes in high- and low-risk groups in TCGA and GSE84437 cohort. (**A**) TCGA dataset. (**B**) GSE84437 dataset. *, **, *** and **** represent p < 0.05, p < 0.01, p < 0.001 and p < 0.0001, respectively.

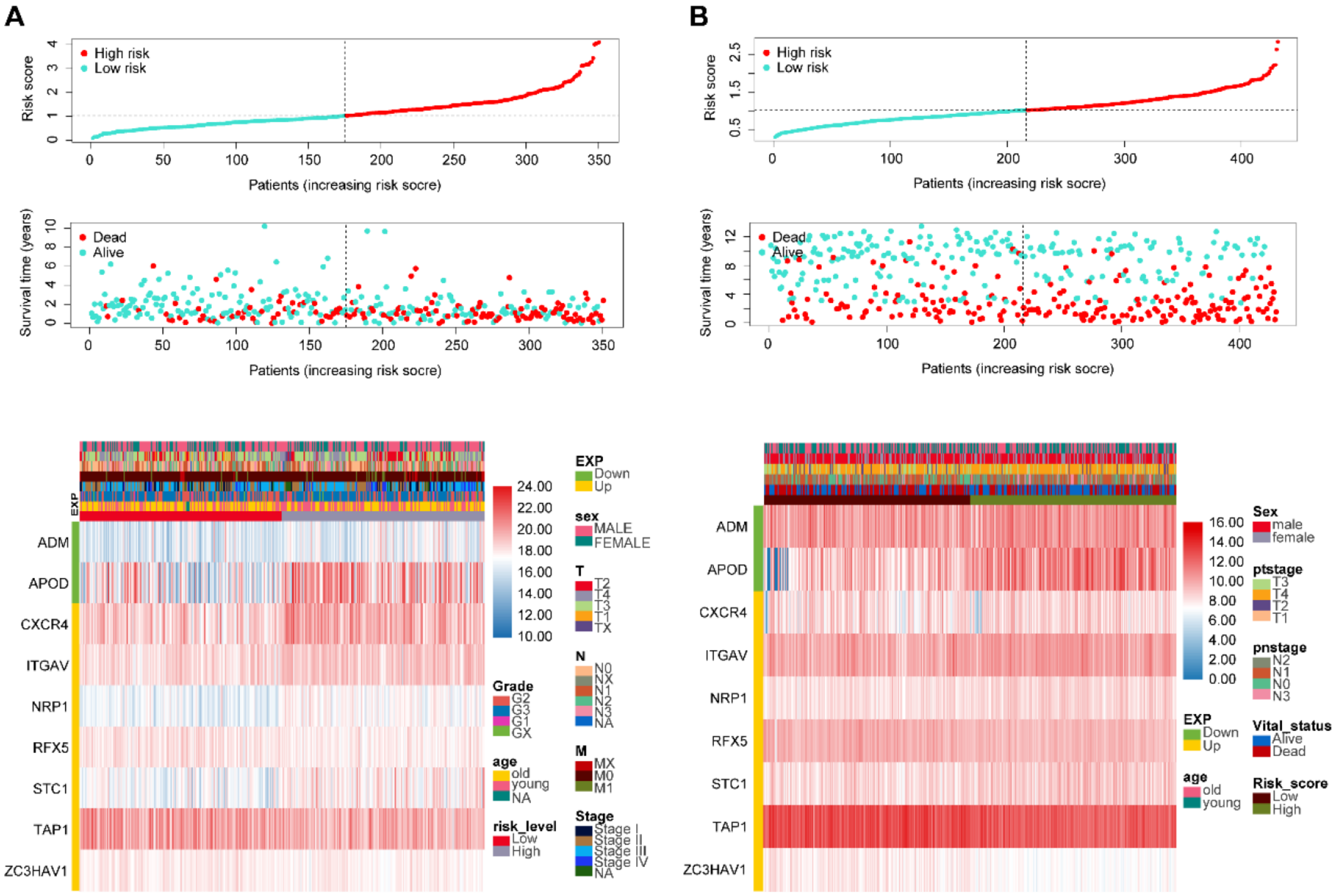

Supplementary Figure 3. The distribution of risk score, survival status, and gene expression panel. Correlation between the prognostic signature and the overall survival of patients in the TCGA (**A**) and GEO (**B**) cohort. The distribution of risk scores (upper), survival time (middle), and IRGS expression levels (lower). The black dotted lines represent the median risk score cut-off dividing patients into low- and high-risk score groups. The red dots and lines represent the patients in the high-score groups. The green dots and lines represent the patients in the low-score groups.

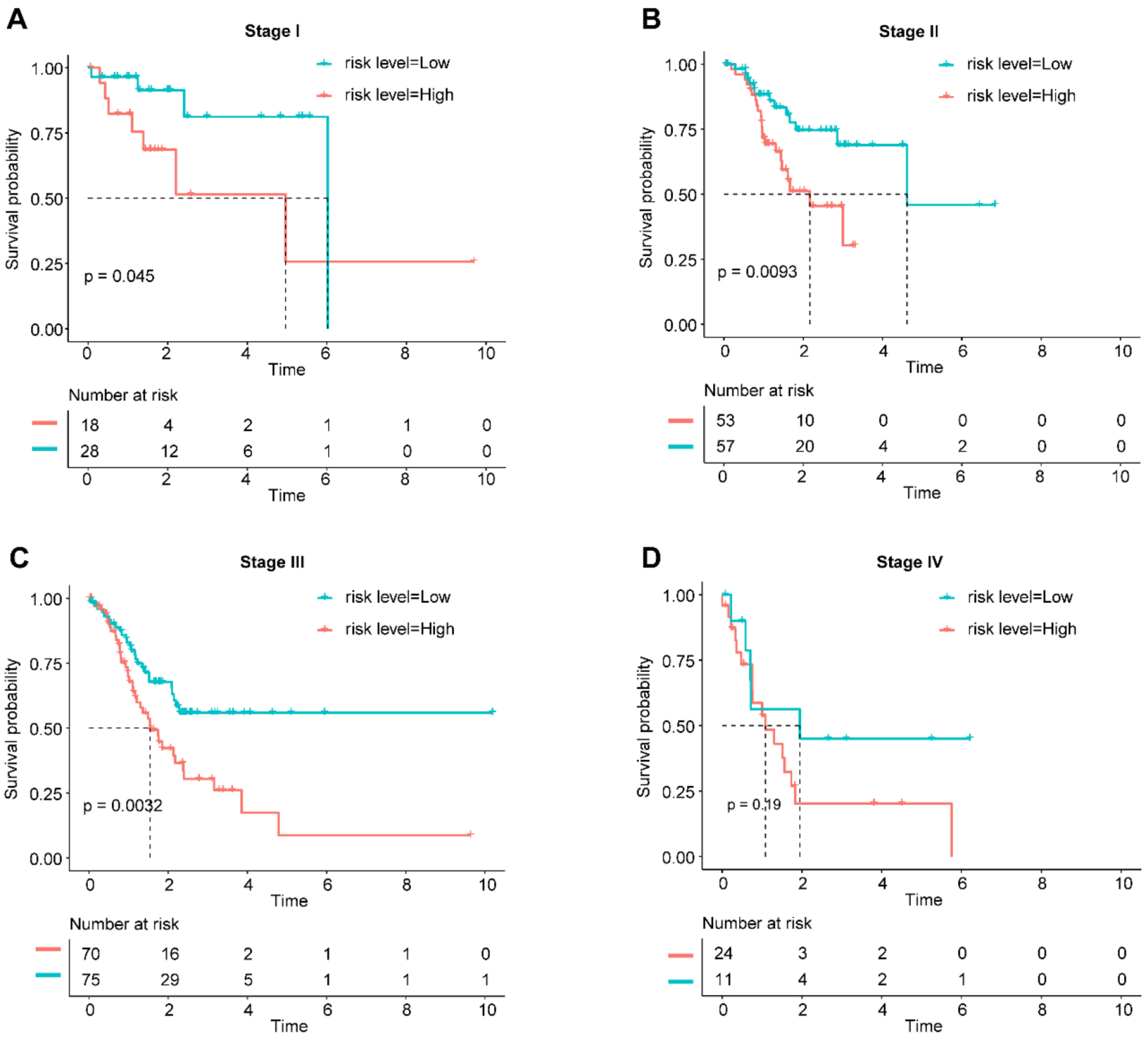

Supplementary Figure 4. Kaplan-Meier curves of OS in different stages (**A**–**D**) of GAC based on risk score, respectively.

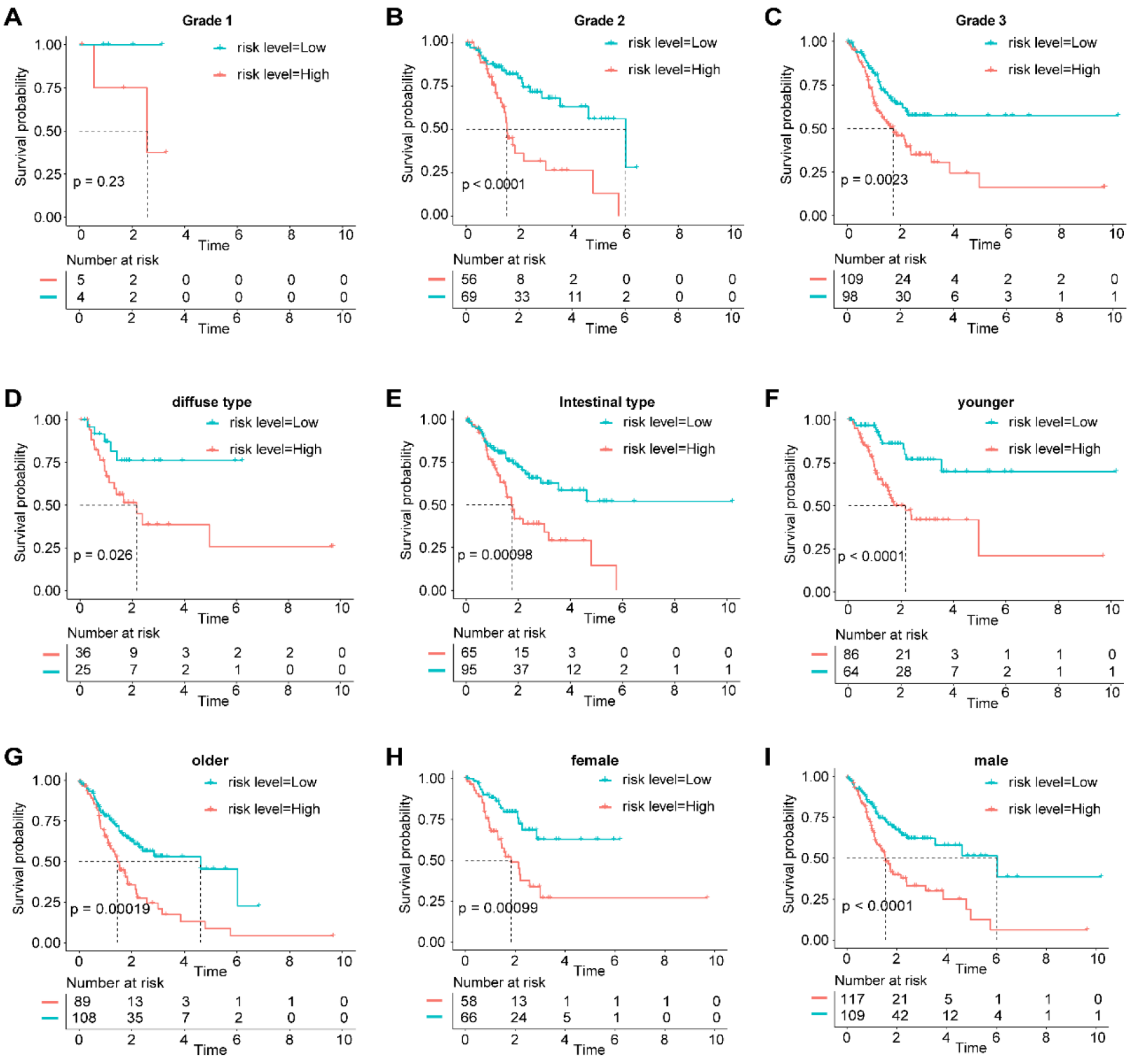

Supplementary Figure 5. Kaplan-Meier curves of OS in the different clinical subtype of GAC based on risk score, respectively. (**A**–**C**) Kaplan-Meier curves of OS in different grades of differentiation of GAC based on risk score, respectively. (**D**, **E**) Kaplan-Meier curves of OS in different histological phenotypes of GAC based on risk score, respectively. (**F**, **G**) Kaplan-Meier curves of OS in different age of GAC based on risk score, respectively. (**H**, **I**) Kaplan-Meier curves of OS in different sex of GAC based on risk score, respectively.

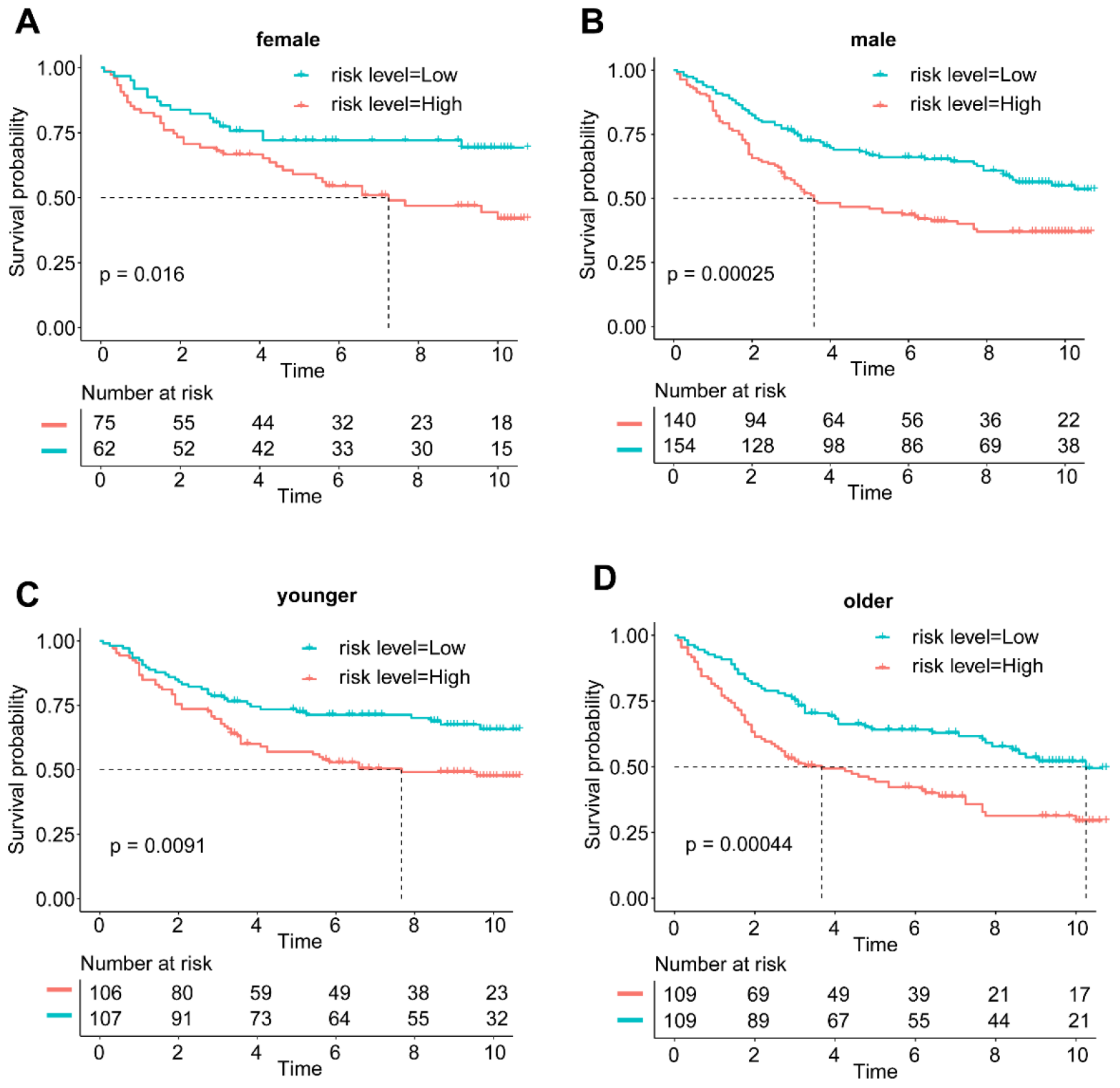

Supplementary Figure 6. Survival analysis of all GAC patients stratified by gender and age in the GSE84437 cohort. Kaplan-Meier curves of OS in female (**A**), male (**B**), younger (**C**), and older (**D**) patients based on risk score in GAC population.

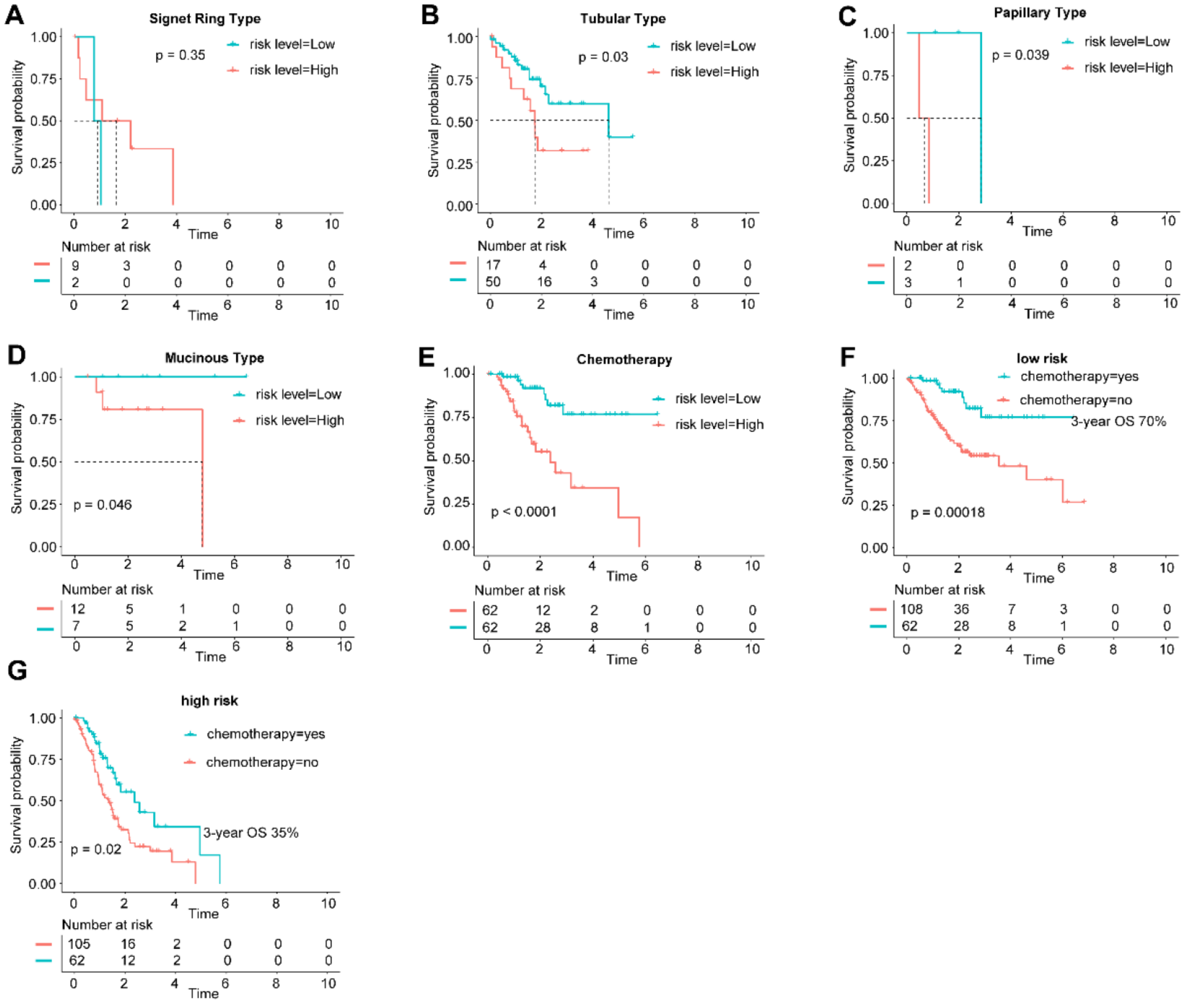

Supplementary Figure 7. Survival analysis of all GAC patients stratified by different histological phenotypes (**A**–**D**) of WHO standards in the TCGA cohort. The effect of chemotherapy is different between high and low risk groups (**E**–**G**).

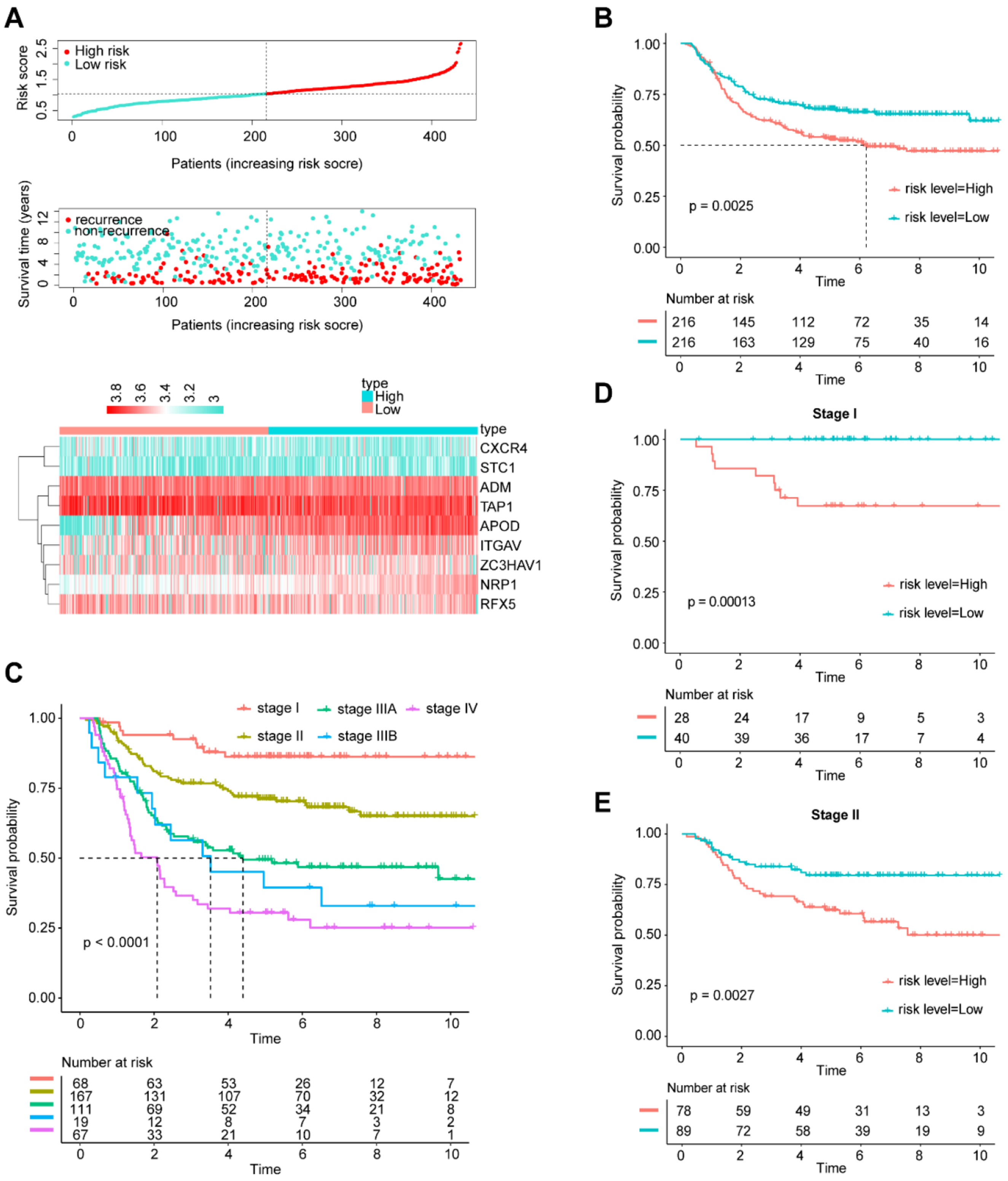

Supplementary Figure 8. Validation of the prognostic performance of immune related gene signature in GSE26253. (**A**) the distribution of risk score, recurrence status, and gene expression panel. The distribution of signature scores (top), recurrence time (middle), and gene expression levels (bottom). The black dotted lines represent the median risk score cut-off dividing patients into the low- and high-risk groups. The red dots and lines represent the patients in the high-score group. The green dots and lines represent the patients in the low-score group. (**B**) Kaplan-Meier curves of RFS in all GAC patients based on the risk score. (**C**) Kaplan-Meier curves of RFS in all GAC patients based on stage. (**D**) Kaplan-Meier curves of RFS in stage I GAC patients based on the risk score. (**E**) Kaplan-Meier curves of RFS in stage II GAC patients based on the risk score.

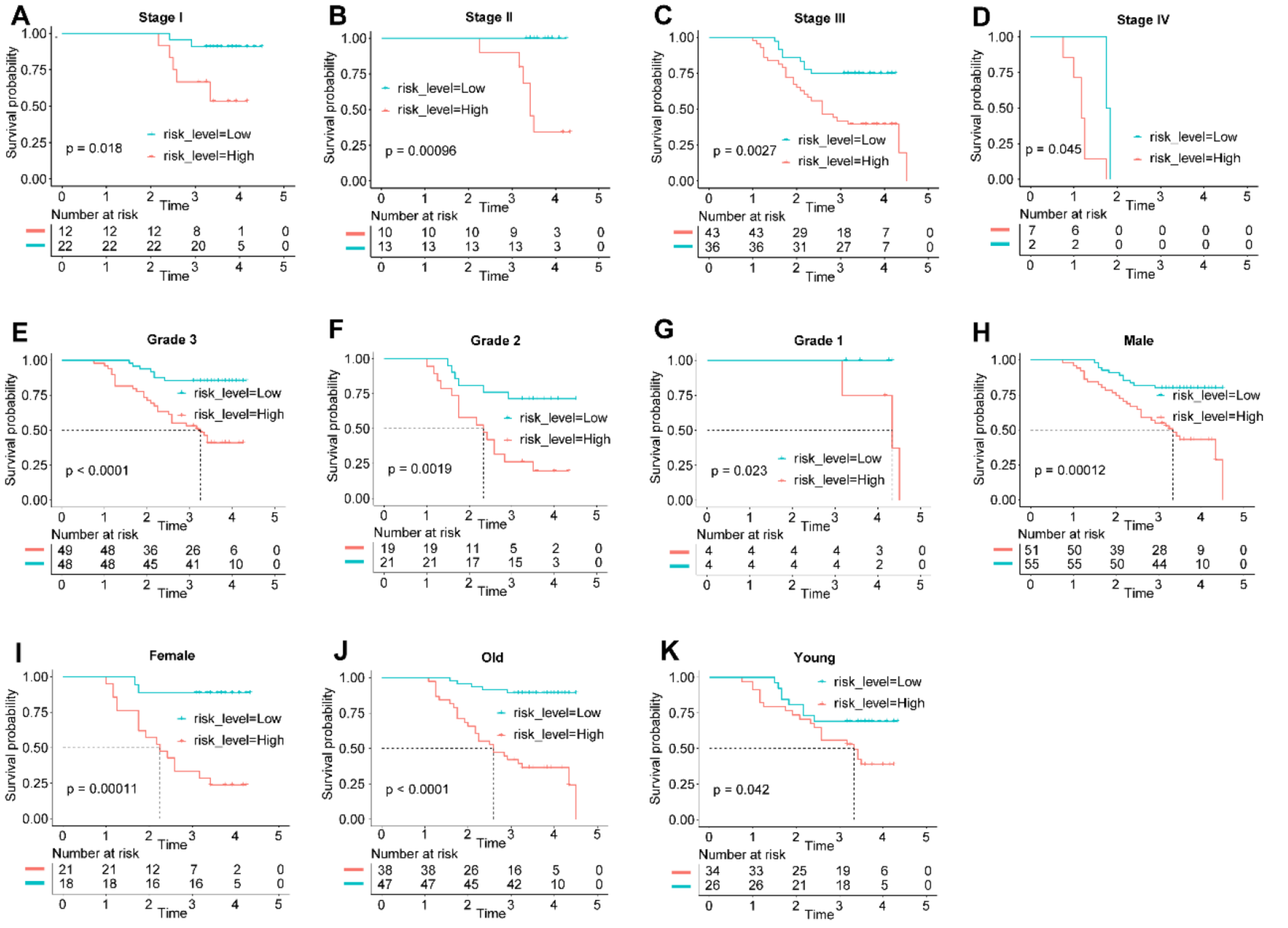

Supplementary Figure 9. Survival analysis of all GAC patients stratified by stage (**A**–**D**), grade (**E**–**G**), gender (**H**, **I**), and age (**J**, **K**) in the independent cohort.

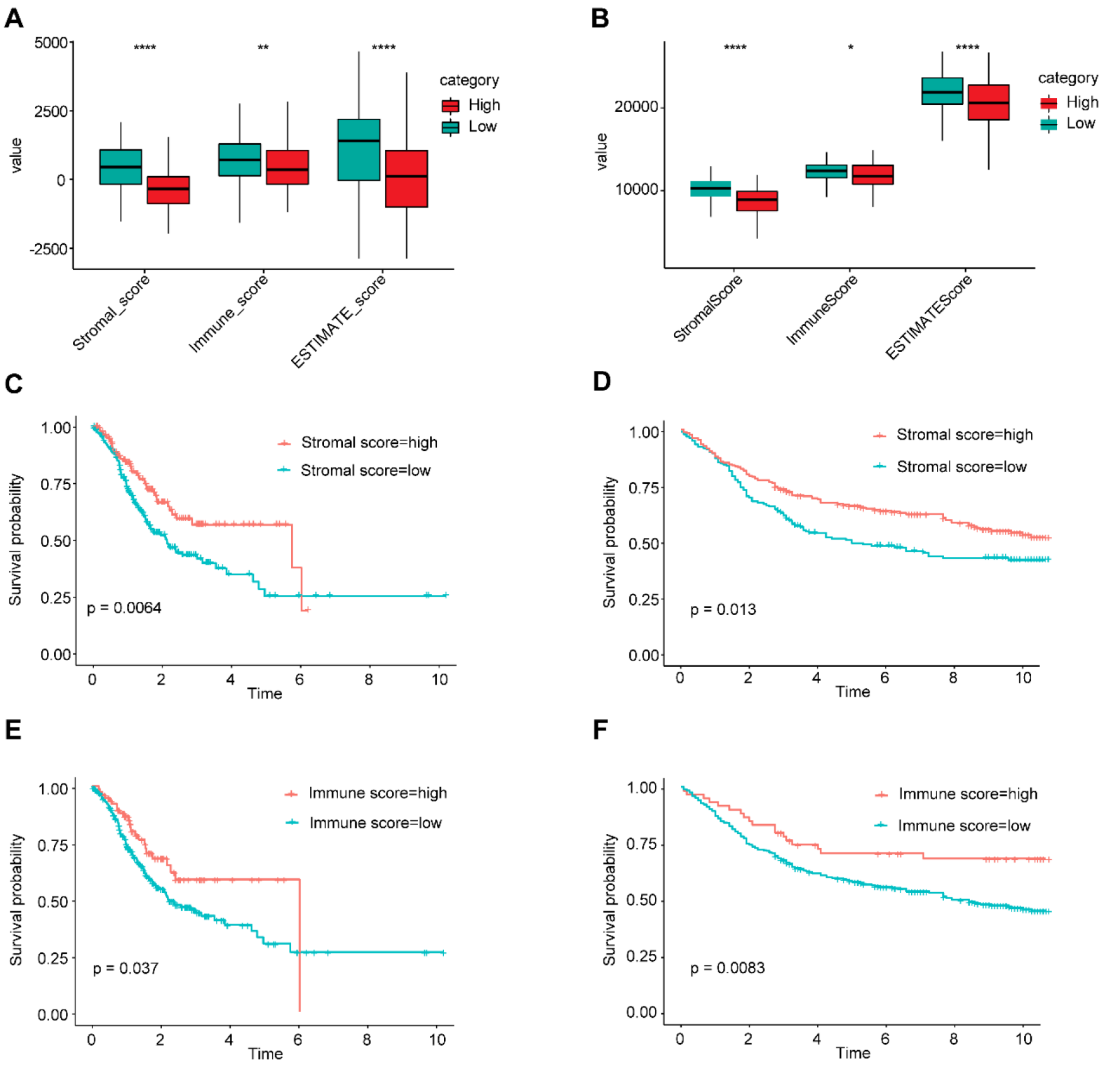

Supplementary Figure 10. The relationship between immune immersion scores and risk scores and prognosis. (**A**, **B**) Differences in the stromal, immune, and ESTIMATE score between high and low risk groups in TCGA and GSE84437 cohorts, respectively. (**C**–**F**) Impact of stromal, immune, and ESTIMATE score on overall survival of GAC based on KM analysis in TCGA and GSE84437 cohorts, respectively. *, **, *** and **** represent $p < 0.05$, $p < 0.01$, $p < 0.001$ and $p < 0.0001$, respectively.

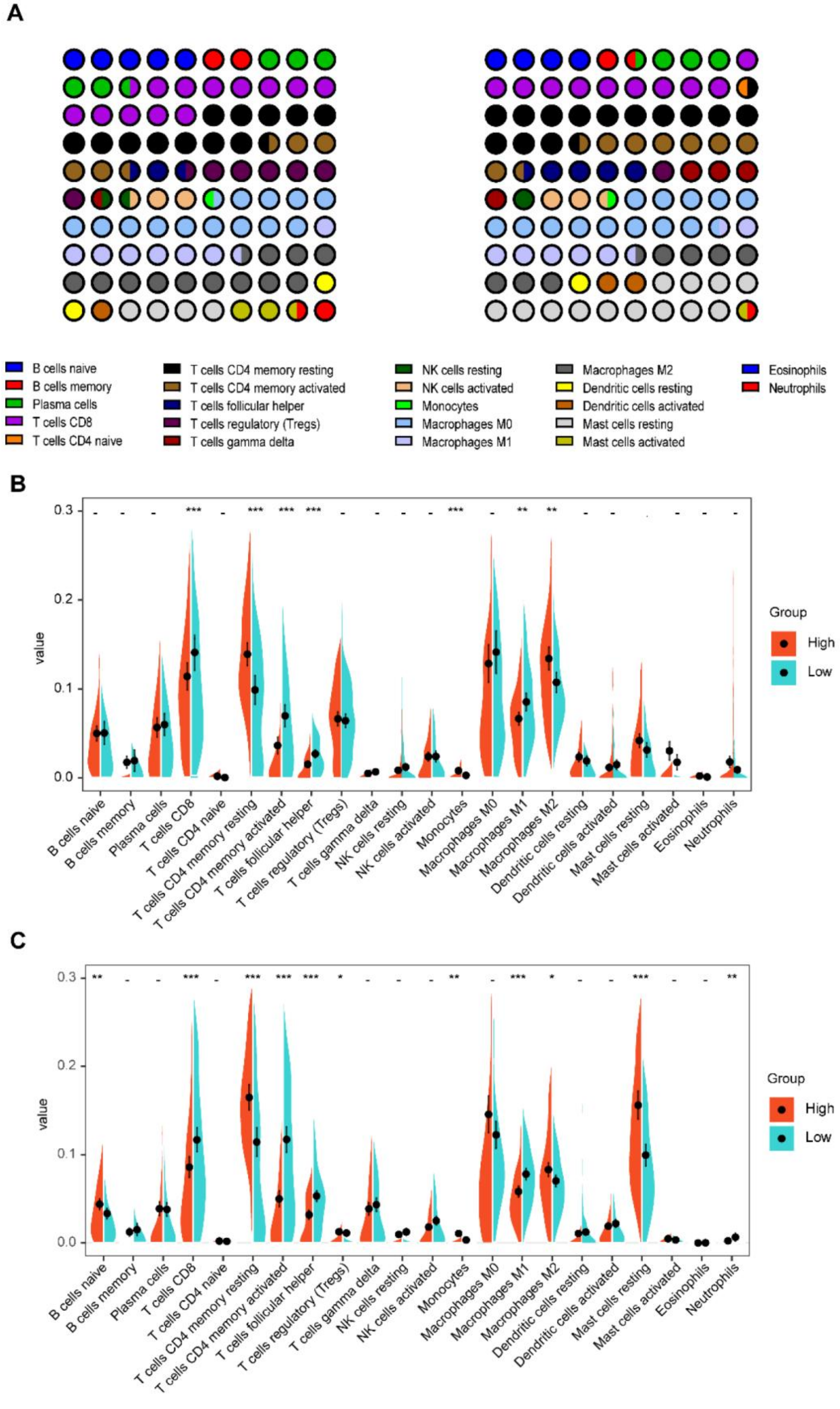

Supplementary Figure 11. The relationship between risk score and immune cell expression in GAC. (**A**) estimated immune cell expression in TCGA and GSE84437, respectively. Different expression of immune cells in high- and low-risk groups in TCGA (**B**) and GSE84437 (**C**), respectively. *, **, *** and **** represent $p < 0.05$, $p < 0.01$, $p < 0.001$ and $p < 0.0001$, respectively.

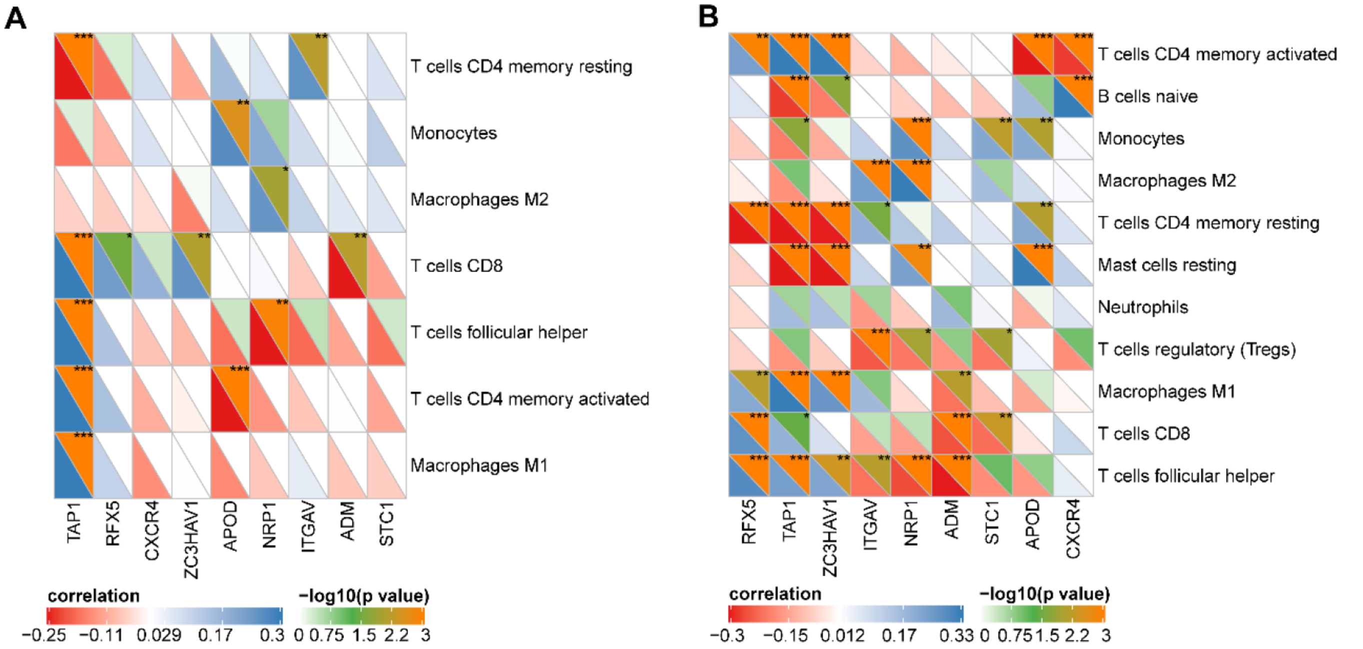

Supplementary Figure 12. The Pearson correlation coefficients of 9 IRGS with various differential expressed immune cells in TCGA and GSE84437 cohort. (**A**) TCGA dataset. (**B**) GSE84437 dataset. *, **, *** and **** represent p < 0.05, p < 0.01, p < 0.001 and p < 0.0001, respectively.

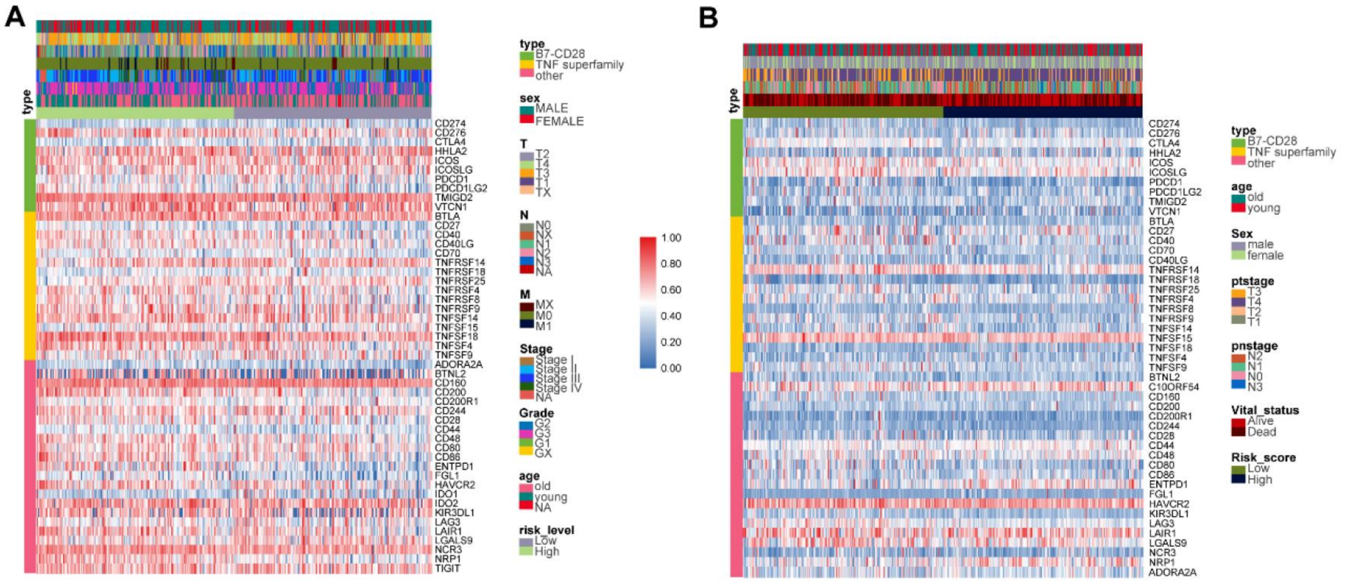

Supplementary Figure 13. The expression profile of costimulatory/coinhibitory immune checkpoints landscape in TCGA and GSE84437, respectively. (**A**) TCGA; (**B**) GSE84437.

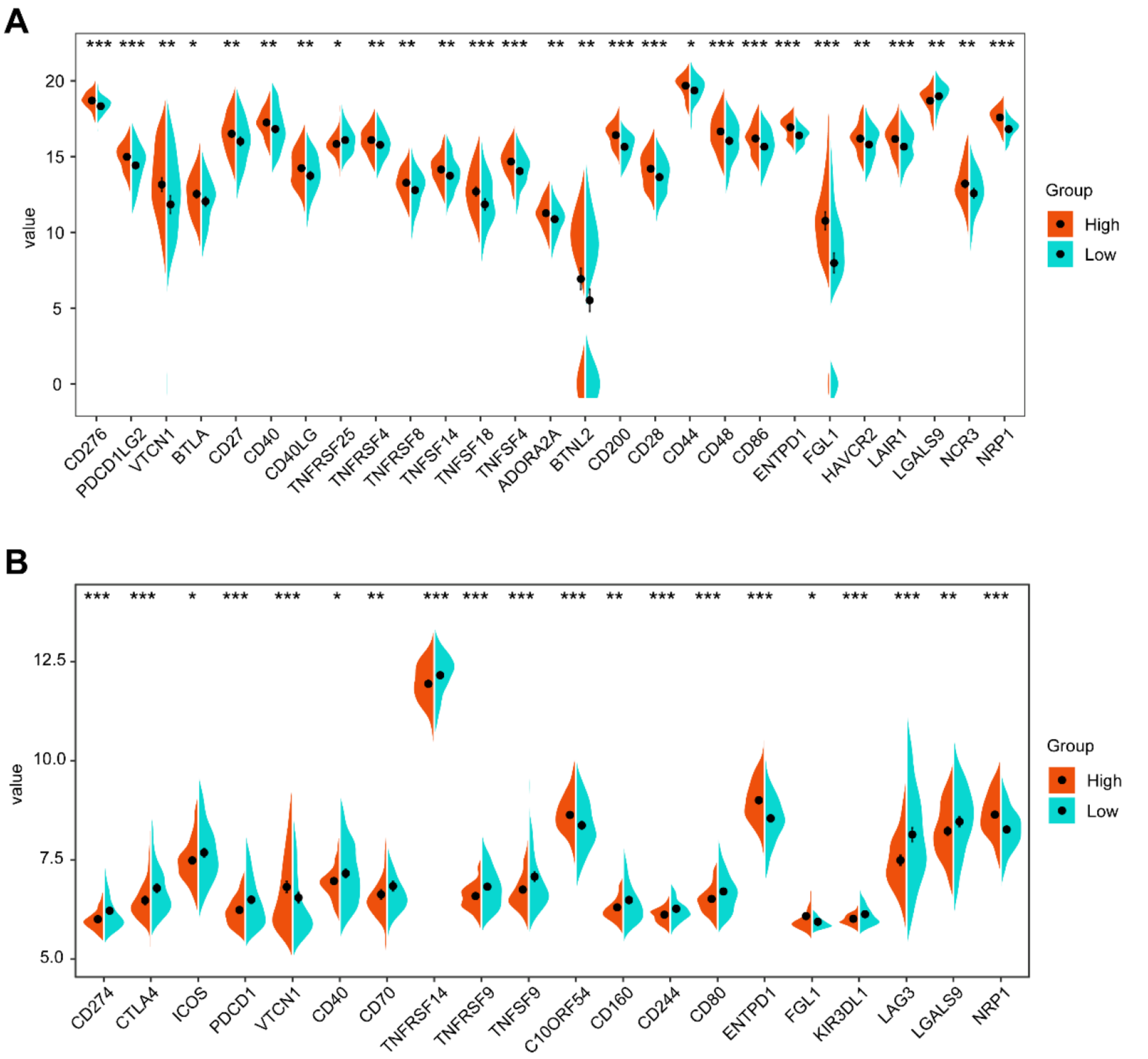

Supplementary Figure 14. Different expression of immune checkpoints in high- and low-risk groups in TCGA and GSE84437, respectively. (**A**) TCGA dataset. (**B**) GSE84437 dataset. *, **, *** and **** represent $p < 0.05$, $p < 0.01$, $p < 0.001$ and $p < 0.0001$, respectively.

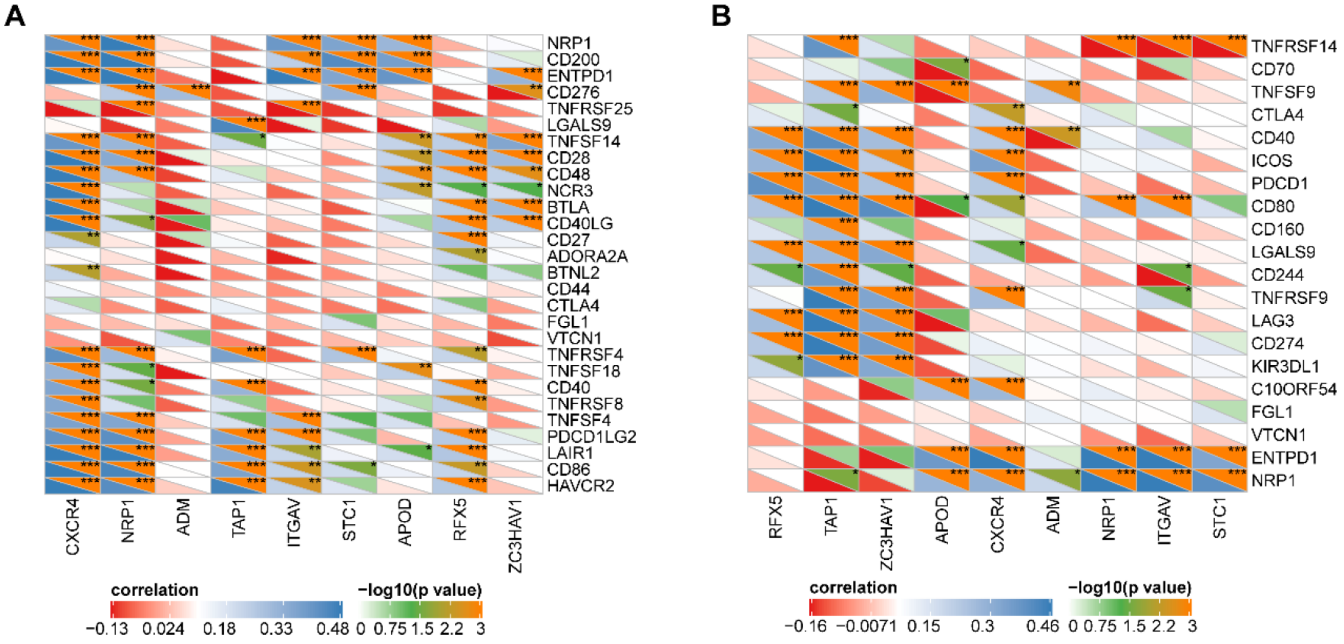

Supplementary Figure 15. The Pearson correlation coefficients of 9 IRGS with various differential expressed immune checkpoints in TCGA and GSE84437 cohort. (**A**) TCGA dataset. (**B**) GSE84437 dataset. *, **, *** and **** represent p < 0.05, p < 0.01, p < 0.001 and p < 0.0001, respectively.

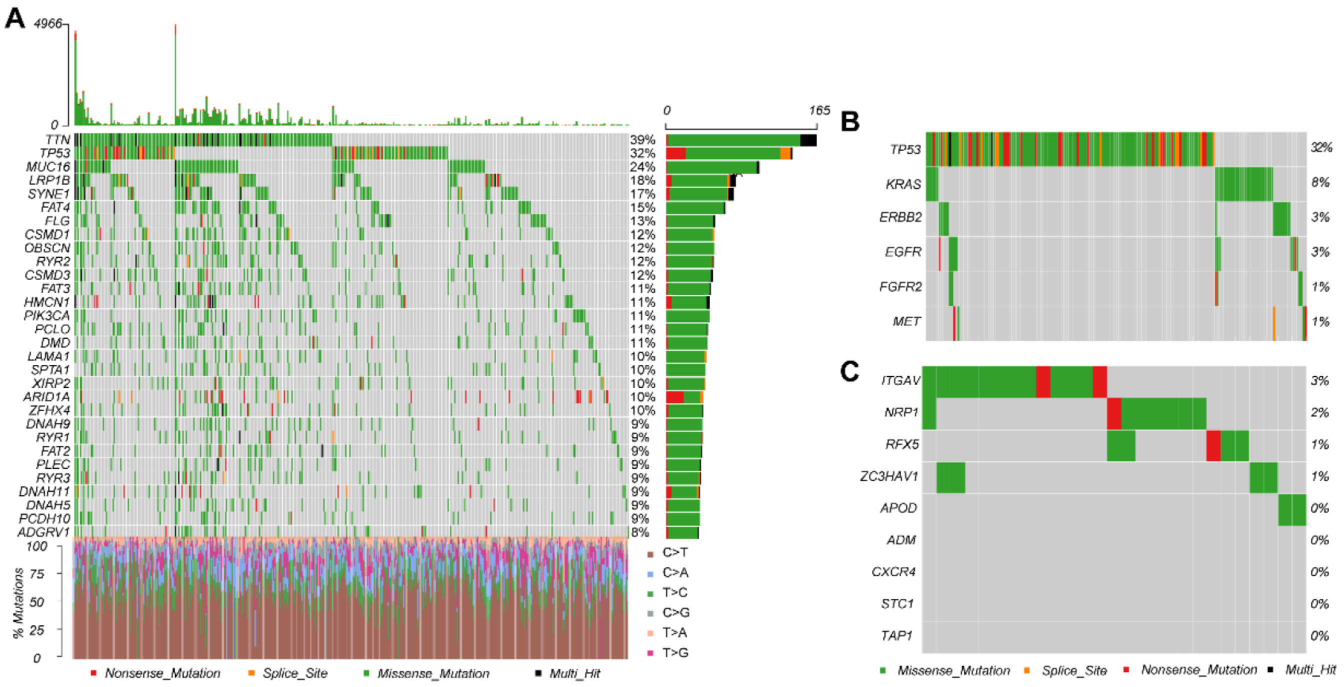

Supplementary Figure 16. Tumor somatic mutation landscape of the IBPS. (**A**) The top 30 of gene mutations in the TCGA-STAD cohort. (**B**) The mutations of the commonly mutant genes TP53, ERBB2, EGFR, FGFR2, MET, and KRAS in the TCGA-COAD cohort. (**C**) The mutation landscape of the 9 IGRs in IBPS.

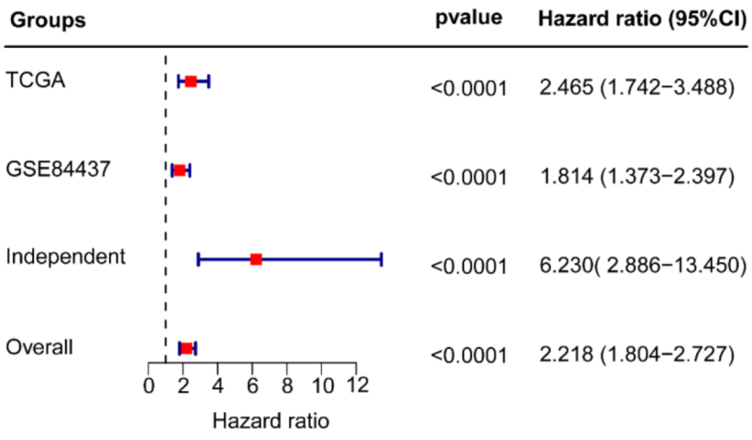

Supplementary Figure 17. Prognostic meta-analysis among three cohorts.

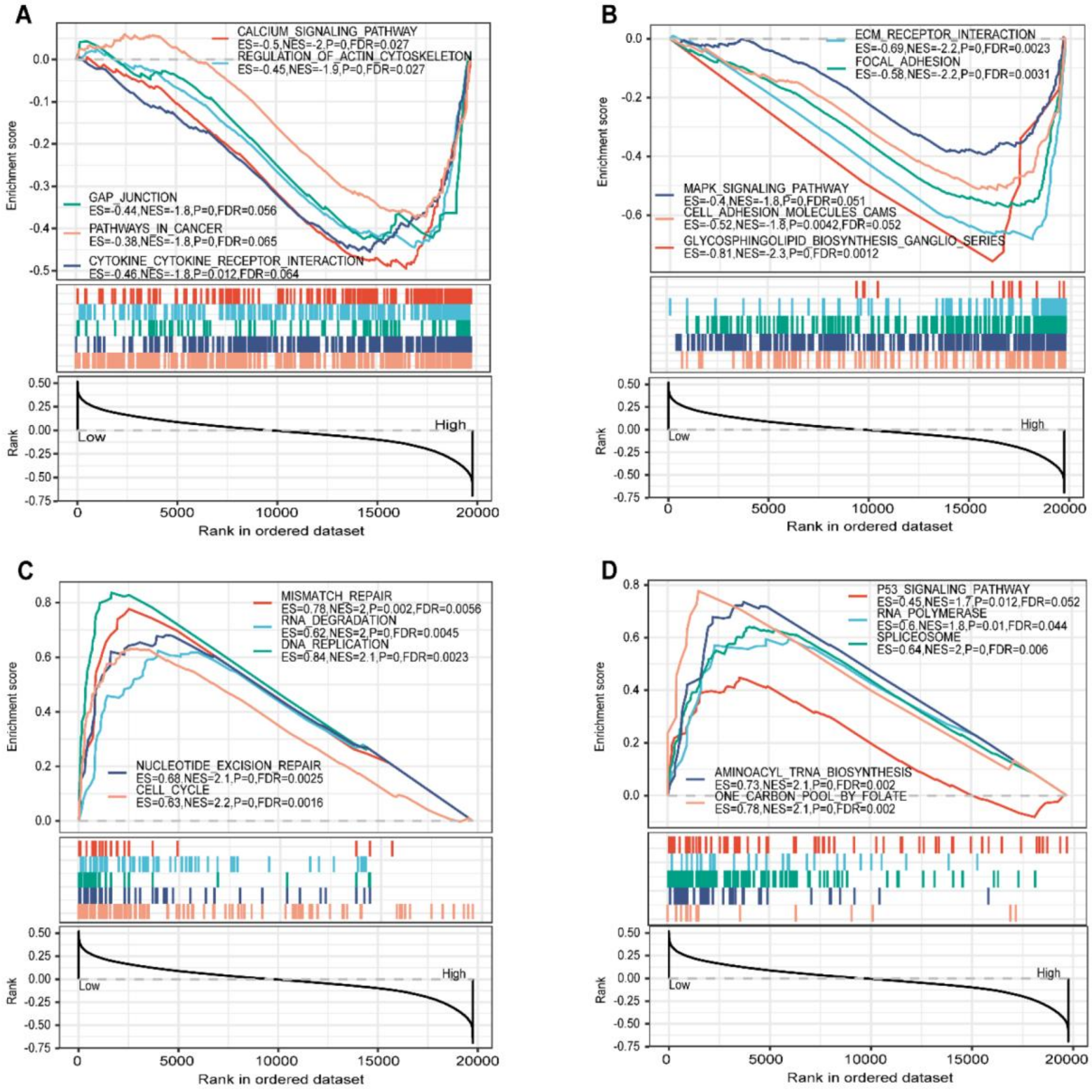

Supplementary Figure 18. GSEA (**A**–**D**). ES, enrichment score; NES, normalized enrichment score; P, P-value; FDR, adjusted P-value.

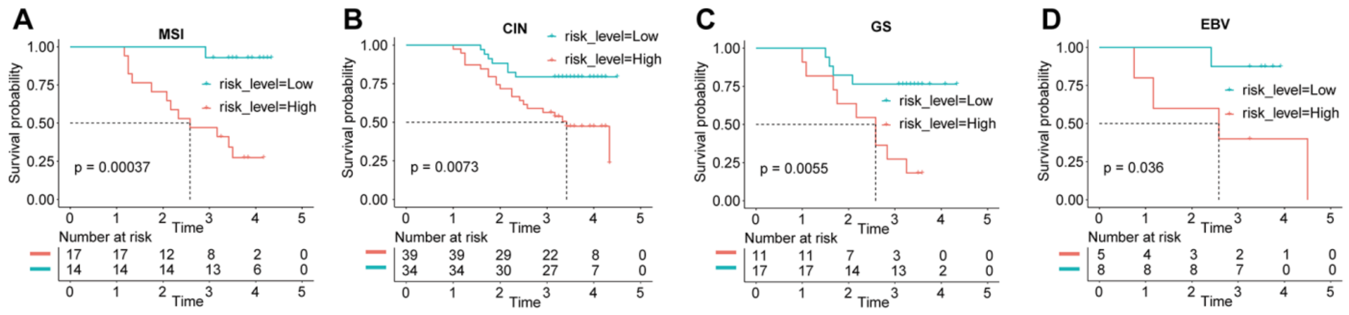

Supplementary Figure 19. Survival analysis of all GAC patients stratified by in different molecular sub-types in the independent cohort. (**A**) Kaplan–Meier curves of overall survival in patients with MSI in the independent cohort. (**B**) Kaplan–Meier curves of overall survival in patients with the chromosome unstable (CIN) type in the independent cohort. (**C**) Kaplan–Meier curves of overall survival in patients with the genomic stable (GS) type in the independent cohort. (**D**) Kaplan–Meier curves of overall survival in patients with EBV in the independent cohort.

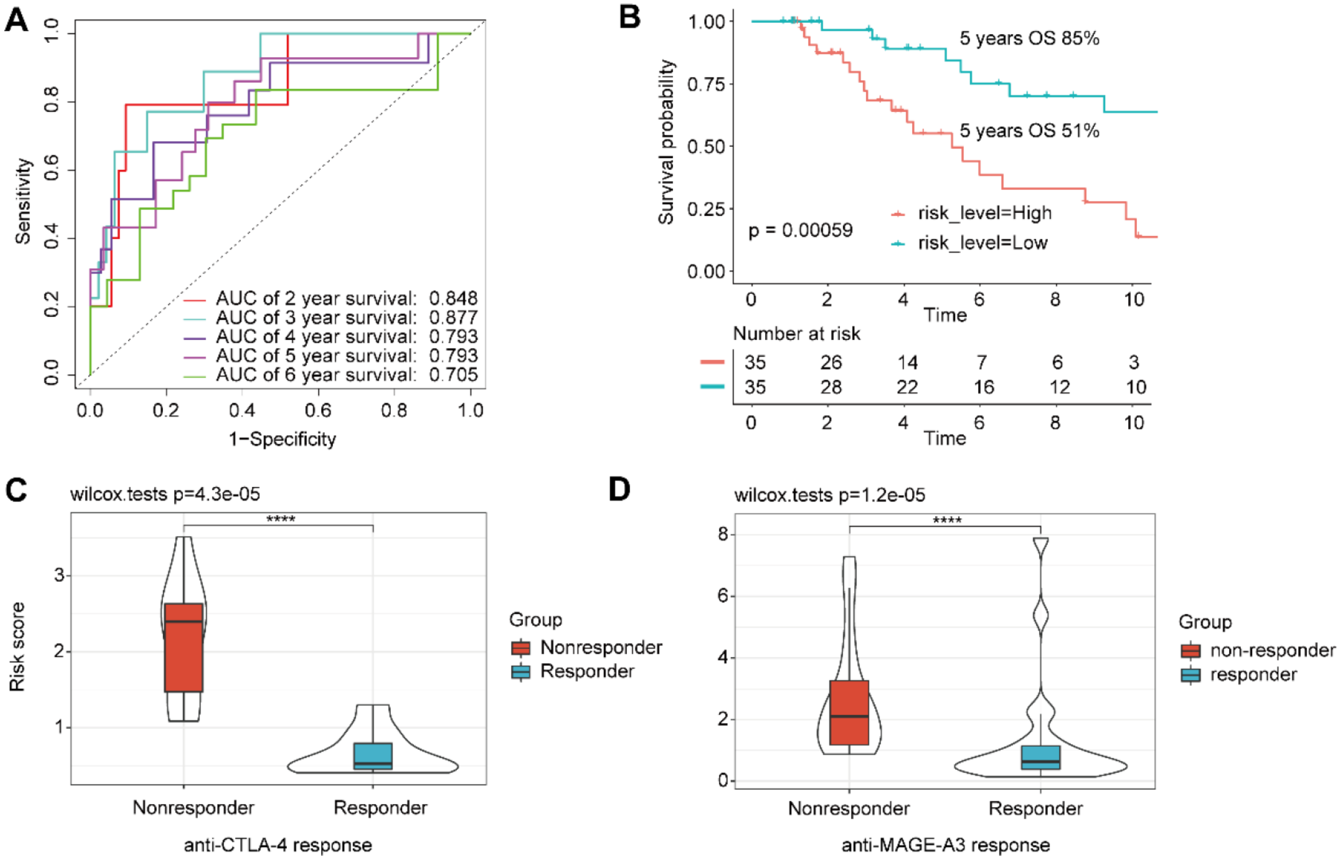

Supplementary Figure 20. Application of signature in immunotherapy. (**A**) Receiver operating characteristic (ROC) curves for signature in the TCGA-SKCM cohort. (**B**) Kaplan–Meier curves of overall survival of patients treated with immunotherapy in TCGA-SKCM cohort. (**C**) Risk score in patients with response (blue) versus those without response(red) (Wilcoxon P =4.3e^{-05}) to anti-CTLA-4 treatment. (**D**) Risk score in patients with response (blue) versus those without response(red) (Wilcoxon P =1.2e^{-05}) to anti-MAGE-A3 treatment.2.
