# Supplementary material for: Clinical significance and prognostic role of an immune-related gene signature in gastric adenocarcinoma
Source: Aging (Albany NY). 2021 Jul 11;13(13):17734–67. doi: 10.18632/aging.203266 (PMC8312416; doi:10.18632/aging.203266)
Supplement: Supplementary Tables [file aging-13-203266-s002.pdf]

SUPPLEMENTARY TABLES

Supplementary Table 1. Primer sequences for qRT-PCR.

Gene name	Forward primer	Reverse primer	bp
ADM	5'- TGGGTTCGCTCGCCTTCCTAG-3'	3'- ACATCCGCAGTTCCCTCTTCCC-5'	114
APOD	5'- TGCTGCTGCTGCTGCTTTCC-3'	3'- ACCGGAGGATTGGGGCACTTC-5'	91
CXCR4	5'- ACCTCTACAGCAGTGTCCTCATCC-3'	3'- GATCCAGACGCCAACATAGACCAC-5'	131
ITGAV	5'- TGTGGCTGTCGGAGATTTCAATGG-3'	3'- TTCCCAAAGTCCTTGCTGCTCTTG -5'	80
NRP1	5'- CTCCCGCCTGAACTACCCTGAG -3'	3'- CCCGACAGCCGTGACAAAGC-5'	106
RFX5	5'- CACCTGGAAGAGCACACTGACAC -3'	3'- GGCGGCAACAGGCAAGACTC -5'	91
STC1	5'- CCATGAGGCGGAGCAGAATGAC -3'	3'- GCCGACCTGTAGAGCACTGTTG -5'	106
TAP1	5'- TACCGCCTTCGTTGTCAGTTATGC -3'	3'- GAAGCCGACGCACAGGGTTTC -5'	119
ZC3HAV1	5'- AGGCTCGTCCAAGGCTACTGATC -3'	3'- AGGTCCTCTTGACTGCCGTTCTC -5'	87
GAPDH	5'- GAAAGCCTGCCGGTGACTAA -3'	3'- GCCCAATACGACCAAATCAGAG -5'	150

qRT-PCR, quantitative real-time polymerase chain reaction.

Supplementary Table 2. The 9 genes in prognostic model in TCGA cohort.

	β	HR	lower .95	upper .95	z Pr (>|z|)
ADM	0.0622	1.0642	0.9079	1.2473	0.4428
APOD	0.0771	1.0801	0.9939	1.1739	0.0695
CXCR4	0.1335	1.1428	0.9603	1.3598	0.1326
ITGAV	0.2369	1.2674	0.9774	1.6434	0.0739
NRP1	0.0968	1.1016	0.8458	1.4347	0.4730
RFX5	-0.2405	0.7862	0.5410	1.1426	0.2073
STC1	0.0531	1.0546	0.8820	1.2609	0.5601
TAP1	-0.0892	0.9147	0.7624	1.0974	0.3371
ZC3HAV1	-0.5188	0.2146	0.3908	0.9066	0.0157

Supplementary Table 3. Univariable and multivariable Cox regression analysis of IBPS and characteristics with RFS in GSE26253 cohort.

Variable	Recurrence free survival					
	Univariate cox			Multivariate cox		
	p value	HR	95%*CI*	*p* value	HR	95%*CI*
**TNM stage**						
IB						
II	0.0097	2.5480	1.2540-5.1770	0.0117	2.4890	1.2249-5.0590
IIIA	<0.0001	5.1090	2.5280-10.3240	<0.0001	4.8790	2.4104-9.8740
IIIB	<0.0001	6.5320	2.7510-15.5090	<0.0001	5.7740	2.4119-13.8230
IV	<0.0001	9.0410	4.4290-18.4560	<0.0001	8.5870	4.1984-17.5630
**Risk score**						
Increasing	0.0027	1.5840	1.1730-2.1400	0.0310	1.3520	1.1020-1.8340

IBPS, immune gene set-based prognostic signature; HR, hazard ratio; CI, confidence interval.

Supplementary Table 4. Univariable and multivariable Cox regression analysis of IBPS and characteristics with OS in independent cohort.

Variable	Overall survival					
	Univariate cox			Multivariate cox		
	p value	HR	95%*CI*	*p* value	HR	95%*CI*
**Age**						
≥62 VS <62	0.3390	1.2900	0.7138-2.3320	0.6621	1.1447	0.6244-2.0980
**Sex**						
Male VS Female	0.8730	1.0590	0.5232-2.1440	0.6137	0.8244	0.3894-1.7450
**TNM stage**						
I						
II	0.6696	1.3100	0.3791-4.5250	0.7762	1.1974	0.3457-4.1470
III	0.0183	3.1480	1.2144-8.1610	0.0794	2.3727	0.9053-6.2310
IV	<0.0001	57.4890	15.4370-214.1000	<0.0001	50.6699	12.1610-211.1100
**Grade**						
1						
2	0.4270	1.3520	0.6429-2.8420	0.4571	1.3361	0.6226-2.8670
3	0.2600	1.5230	0.7326-3.1670	0.3546	1.4539	0.6583-3.2110
**Risk score**						
Increasing	<0.0001	6.2300	2.8860-13.4500	<0.0001	6.0282	2.6909-13.5040

IBPS, immune gene set-based prognostic signature; HR, hazard ratio; CI, confidence interval.
